# Supplementary material for: Allosteric regulation and crystallographic fragment screening of SARS-CoV-2 NSP15 endoribonuclease
Source: Nucleic Acids Res. 2023 Apr 28;51(10):5255–70. doi: 10.1093/nar/gkad314 (PMC10250223; doi:10.1093/nar/gkad314)
Supplement: gkad314_Supplemental_Files [file gkad314_supplemental_files.zip › Supplementary datasets.zip!/81/Refine_9/01-BUSTER/Cycle-2/LIST.html]

Refining

**BUSTER 2.X**: A suite of programs for macromolecular structure refinement by maximum-likelihood and Bayesian methods  
Copyright © 1992-2020 Global Phasing Ltd.  
All rights reserved.  
Authors: G. Bricogne, E. Blanc, M. Brandl, C. Flensburg, P. Keller, W. Paciorek, P. Roversi, O. Smart, C. Vonrhein, T. Womack  

Please reference use of the software as:

- BUSTER-TNT 2.X, Global Phasing Ltd, Sheraton House, Cambridge CB3 0AX, UK

Version: 2.10.3 for linux64-ifort made on Sep 18 2020 at 01:21:52  
Licenced to: Diamond Light Source, Scientific Software  

```
---


### Parsing of the BUSTER cardfile

TITLE : autoBUSTER refinement

   Cell information

   Symmetry information

 Using CCP4 X-ray form factors.

   Missing structure chemical composition

 Using ATOM block for chemical composition.


   Shell information

### Parsing of the TNT cardfile


---


### Reading observations file

 Number of reflexions in input:  57108

      Number of accepted reflexions:  55835

      Number of rejected reflexions:      0

   Reflexions in test set:            2859

   Reflexions in working set:        52976


---


### 1. Generating initial model

 2.10 * Shannon sampling rate. NX NY NZ:   288  288  240


  Effective Shannon rates are:    2.29    2.29    2.67


   Allocated BUFFER of size:     76.465 Mb.

   Initial Geometry Information


 No missing atoms declared


   Summary of model volumes

 No missing atoms declared: no prior computed


 Computing bulk solvent model scattering


 NCSMASK PDB radius for solvent mask:     2.15 Angstrom

 running PDBSET (setting cell and spacegroup P1) ... done
 running NCSMASK [1] (creating mask, SMOOTH 1.05084) ... done
 running MAPDUMP (to check if reduction to unit cell is needed) ... done
 running MAPMASK (converting mask to map) ... done

   Generation of babslv by theta-filter

   Electron density in frg mask:  0.420 Å-1

 FOBS  put on rough absolute scale by Wilson plot :
           K        =  0.47314E+02
           B        =    -18.550

 

### Cycle 0001: MaxLik scaling and refinement


   MaxLik Scaling Cycle 0001

   Wilson plot

   Statistics in resolution bins just after scaling
```

|  |  |
| --- | --- |
|  | - Rxpct factors plot- Rxpct factors table- <Log-Lik Gain>- Ln Sigma A- Correlation Coefficients |

```
   <Log-Likelihood> of null-hypothesis

Structure Factor Amplitudes


   Average Figure of Merit

 Switching off refinement of overall temperature factor


 Added B =     0.6733 Å2 to all atoms in fragment


 Added B =     0.6733 Å2 to B_SOLV

```
gelly refinement module
 Authors (2004-2009) O. S. Smart, C. Flensburg, W. Paciorek, C. Vonrhein, 
                     M. Brandl, T. O. Womack and G. Bricogne
         (2010-2011) O. S. Smart, C. Flensburg, W. Paciorek, C. Vonrhein, 
                     T. O. Womack and G. Bricogne
         (2012-2014) O. S. Smart, C. Flensburg, W. Paciorek, C. Vonrhein, 
                     and G. Bricogne
         (2015-2020) G. Bricogne, C. Flensburg, W. Paciorek, C. Vonrhein
 Copyright © 2004-2020 Global Phasing Ltd. 
 All rights reserved.
 gelly module compilation date Sep 18 2020 01:18:35
 
 gelly uses routines from TNT geometry. References: 
     Tronrud, D. E., Ten Eyck, L. F., Matthews, B. W. (1987)
     "An Efficient General-Purpose Least-Squares Refinement Program 
     for Macromolecular Structures". Acta Crystallogr A, 43:489-501.
     Tronrud, D. E. (1997). "The TNT Refinement Package", 
     in Macromolecular Crystallography, Part B, Eds Charlie Carter 
     and Robert Sweet, Methods in Enzymology, 277:306-319.
 wildcard matching function by Julian Robichaux http://www.nsftools.com

 No -random_seed flag found. Random number stream initialised using:
2147483562
2147483398

 Control flags set to:  -autoncs -hydrogens_input_occ2zero \
                     -hydrogens_output_occ2heavy -screen \
                     100 -screen_sigma \
                     3.0 -keeppdb \
                     1000000 -taomonitor_verbose \
                     2 
 

 ############### Starting setup via TNT routines

 Setting up tnt routines including license check:

                   TNT Stereochemistry Program
 Version: 5.22.3.0
 Licence for Diamond Light Source, Scientific Software is valid
 
 Using TNT geometry routines to load the tnt control file: gelly.tnt
 Using TNT routines to load initial coordinates from file fragment.cor
 Expecting 5526 atoms

 Found     2171 "NOTE BUSTER_RESIDUE_ATOM_DATA" cards
 Have copied information for    5526 atoms from TNT to gelly's store 
 
 The TNT WEIGHT cards used by this run:
 -----WEIGHT       BOND     2.0
 -----WEIGHT       ANGLE    2.0
 -----WEIGHT       IMPROPER 0.0
 -----WEIGHT       TORSION  2.0
 -----WEIGHT       PSEUDO   0.0
 -----WEIGHT       TRIGONAL 2.0
 -----WEIGHT       PLANE    5.0
 -----WEIGHT       CONTACT  5.0
 -----WEIGHT       BCORREL  10.0
 -----WEIGHT       CHIRAL   5.0
 -----WEIGHT       NCS      50.0
 -----WEIGHT       IDEAL    4.0
 -----WEIGHT       RFACTOR  2.19
 
 Dictionary versions picked up from NOTE BUSTER_RESTRAINT_DICT cards as: 
 ------ protgeo_eh99.dat (V1.6.4.17) 20191001  STANDARD AMINO ACID DICTIONARY. BONDS AND ANGLES FROM ENGH AND HUBER EH99. OTHER VALUES BASED ON PREVIOUS TNT OR TAKEN FROM CCP4. INCLUDES HYDROGEN ATOMS.
 ------ exoticaa.dat (V1.3.4.15) 20200327  COLLECTION OF NON-STANDARD AMINO ACIDS, MAINLY EH91 WITHOUT IDEAL DISTANCE INFO
 ------ nuclgeo.dat (V1.13.4.8) 20190909
 ------ bcorrel.dat (V1.15.10.2) 20190616
 ------ contact.dat (V1.15.12.10) 20191121
 ------ idealdist_contact.dat (V1.3.4.12) 20191001  IDEAL-DISTANCE CONTACT TERM DATA AS USED IN PROLSQ. VALUES USED HERE ARE BASED ON DATA HARVESTING OF PDB AS OF 20190109.
 ------ restraints for LIG (Unknown) from cif dictionary Z1741794237.cif
 ------ assume.dat (V1.9.4.2) 20180705
 
 Have picked up spacegroup name for pdb output as 'P 63      '
 Setting up Chain/Residue arrays
 
 Have command line specification -autoncs*
     Have found       1 NCS relations
     Pairwise matching residue number and type between chains: 
                    A        B    
          A         .      100%  
          B                  .   
       Table key--                                                       
          all   : two chains have 100% match and same number of residues 
         multi  : ditto and they contribute to a multi chain ncs cluster 
          ***%  : two chains have >80% matching residue number/type      
           -    : chains appear to be unrelated                          
      chain A     have identified ncs to     1 other chains. So will weight each autoncs by (       6.666/#)=      6.6660
      chain B     have identified ncs to     1 other chains. So will weight each autoncs by (       6.666/#)=      6.6660
     For .Gelly cards produced see below - after pruning
 
 Setting up BUSTER_SET groups
 Have found    89 BUSTER_SET cards
 Now silently applying automatic/dictionary set definitions. For a verbose listing use '-verbose_set' flag.
 Applying user defined set definitions:
 =====NOTE BUSTER_SET ncsautoXcld = Water --> set 'ncsautoXcld' now has     0 atoms 
 =====NOTE BUSTER_SET ncsautoABset = Chain_A \ ncsautoXcld --> set 'ncsautoABset' now has  2768 atoms 
 Have defined    47 sets: 
      set:                 'All' has   5526 atoms in    699 residues - automatically defined
      set:               'Empty' has      0 atoms in      0 residues - automatically defined
      set:            'ExoticAA' has      0 atoms in      0 residues - defined by dictionary file
      set:           'ExoticNUC' has      0 atoms in      0 residues - defined by dictionary file
      set:              'NotHET' has   5516 atoms in    698 residues - defined by dictionary file
      set:             'Chain_A' has   2768 atoms in    350 residues - automatically defined
      set:             'Chain_B' has   2758 atoms in    349 residues - automatically defined
      set:               'Water' has      0 atoms in      0 residues - defined by dictionary file
      set:          'StdProtein' has   5516 atoms in    698 residues - defined by dictionary file
      set:             'Protein' has   5516 atoms in    698 residues - defined by dictionary file
      set:                'Back' has   2794 atoms in    698 residues - defined by dictionary file
      set:                'Side' has   2722 atoms in    652 residues - defined by dictionary file
      set:        'RnaCanonical' has      0 atoms in      0 residues - defined by dictionary file
      set:        'DnaCanonical' has      0 atoms in      0 residues - defined by dictionary file
      set:     'RnaDnaCanonical' has      0 atoms in      0 residues - defined by dictionary file
      set:     'DnaRnaCanonical' has      0 atoms in      0 residues - defined by dictionary file
      set:         'RnaExtended' has      0 atoms in      0 residues - defined by dictionary file
      set:         'DnaExtended' has      0 atoms in      0 residues - defined by dictionary file
      set:      'RnaDnaExtended' has      0 atoms in      0 residues - defined by dictionary file
      set:      'DnaRnaExtended' has      0 atoms in      0 residues - defined by dictionary file
      set:                 'Dna' has      0 atoms in      0 residues - defined by dictionary file
      set:                 'Rna' has      0 atoms in      0 residues - defined by dictionary file
      set:              'RnaDna' has      0 atoms in      0 residues - defined by dictionary file
      set:              'DnaRna' has      0 atoms in      0 residues - defined by dictionary file
      set:             'Nucleic' has      0 atoms in      0 residues - defined by dictionary file
      set:             'Polymer' has   5516 atoms in    698 residues - defined by dictionary file
      set: 'BufferWeichenberger' has      0 atoms in      0 residues - defined by dictionary file
      set:    'BufferAdditional' has      0 atoms in      0 residues - defined by dictionary file
      set:              'Buffer' has      0 atoms in      0 residues - defined by dictionary file
      set:         'MetalAlkali' has      0 atoms in      0 residues - defined by dictionary file
      set:  'MetalAlkalineEarth' has      0 atoms in      0 residues - defined by dictionary file
      set:     'MetalTransition' has      0 atoms in      0 residues - defined by dictionary file
      set: 'MetalPostTransition' has      0 atoms in      0 residues - defined by dictionary file
      set:    'MetalLanthanides' has      0 atoms in      0 residues - defined by dictionary file
      set:               'Metal' has      0 atoms in      0 residues - defined by dictionary file
      set:         'SugarAlphaD' has      0 atoms in      0 residues - defined by dictionary file
      set:         'SugarAlphaL' has      0 atoms in      0 residues - defined by dictionary file
      set:               'Sugar' has      0 atoms in      0 residues - defined by dictionary file
      set:               'Other' has     10 atoms in      1 residues - defined by dictionary file
      set:            'Hydrogen' has      0 atoms in      0 residues - defined by dictionary file
      set:         'nonHydrogen' has   5526 atoms in    699 residues - defined by dictionary file
      set:             'OccZero' has      0 atoms in      0 residues - defined by dictionary file
      set:          'OccNonZero' has   5526 atoms in    699 residues - defined by dictionary file
      set:             'OccFull' has   5484 atoms in    696 residues - defined by dictionary file
      set:          'OccNonFull' has     42 atoms in      3 residues - defined by dictionary file
      set:         'ncsautoXcld' has      0 atoms in      0 residues - user specified
      set:        'ncsautoABset' has   2768 atoms in    350 residues - user specified
 No command line arguments '-occ_allhet' or '-occ_restyp' so will not setup automated occcupancy refinement
 
 Interpreting CONSTANT and FREE cards: 
 =====CONSTANT     OCC
 Have found and interpreted      1 CONSTANT cards in the TNT input. 
 Before any COMBINE - Number of atoms with fixed XYZ =     0
 Before any COMBINE - Number of atoms with fixed B   =     0
 Before any COMBINE - Number of atoms with fixed OCC =  5526
 Before any COMBINE - Number of atoms with fixed BIJ =     0
                            cf total number of atoms =  5526
 
 There are no COMBINE cards to be found in the TNT card input 
 
 There are no NOTE BUSTER_COMBINE_RES cards to be found in the TNT card input 

 ############### Working out full connectivity list


 ############### Working out equivalent atoms


 Residue with most atoms given below:
        A|58:N(TRP)         
        A|58:CA(TRP)        
        A|58:CB(TRP)        
        A|58:CG(TRP)        
        A|58:CD1(TRP)       
        A|58:NE1(TRP)       
        A|58:CE2(TRP)       
        A|58:CD2(TRP)       
        A|58:CE3(TRP)       
        A|58:CZ3(TRP)       
        A|58:CH2(TRP)       
        A|58:CZ2(TRP)       
        A|58:C(TRP)         
        A|58:O(TRP)         


 Have no '-equiv_check_plus' argument - so will take atom type into
 account when checking for equivalence of otherwise identical atoms.
 

 ############### Looking at dihedrals


 ############### Looking at chirals

 NOTE BUSTER_CHIRAL_ANGLECUT card found - read value=    -31.00 degs from it
 NOTE BUSTER_CHIRAL_SIGMA card found - read value=      1.70 degs from it
 RANGE allowed for B variables in refinement:   from      3.00000 to    300.00000

 ############### Preparing for PDB output


 ############### Processing extra restraints explicitly defined

 No 'NOTE BUSTER_TARGET' cards or -target?? args found (used to read coordinates files for soft ncs to external)
 No 'NOTE BUSTER_OCCSUM'     cards found - so not using sum of occupancy restraints
 No 'NOTE BUSTER_DISTANCE'   cards found - so not setting up any additional atom-atom distance restraints
 No 'NOTE BUSTER_UTILANGLE'  cards found - so not setting up any additional restraints on any angle
 No 'NOTE BUSTER_UTILTOR'    cards found - so not setting up any additional sinusoidal restraints on torsions
 
 --- Have some similarity definitions 
     Similarity restraints on RMSD use superposition routines from: 
     Coutsias, E.A., Seok, C., Dill, K.A.(2004)
     "Using quaternions to calculate RMSD", J. Comput. Chem., 25:1849-1857 

 Have found    1 'NOTE BUSTER_SIM_DEFINE' cards 
     Maximum number of chains involved in any SIM group (excluding the template)=     1


 ########## Interpreting card: NOTE BUSTER_SIM_DEFINE ncsautoAB ncsautoABset B
     Number of atoms in template set=  2768
     Number of related chains (other than template) in SIM set=    1
     List of chains to be restrained (excluding template)=   B   
     Pairwise RMSD, XYZ in Angstroms, for SIM group: ncsautoAB
                 A         B   
     A         -----     ----- 
     B         0.780     ----- 
     Overall RMSD,XYZ for the SIM group is     0.780 Angstroms
     Pairwise rms deviation of [individual B's minus their set/chain average] in Angs**2
                 A         B         Bavg
     A         -----     -----    102.47
     B         46.40     -----     72.99
     Overall RMSD,B   for the SIM group is    46.397 Angs**2
     The number of common atoms for the above fits:
                 A         B   
     A         -----     ----- 
     B          2758     ----- 
     (compared to the template set that has    2768 atoms in it)
     List of atoms that are absent in one or more chains for this group: 
                                    A    B   
                    C01     401     +    -
                    C02     401     +    -
                    C03     401     +    -
                    N04     401     +    -
                    C05     401     +    -
                    C06     401     +    -
                    N07     401     +    -
                    C08     401     +    -
                    C09     401     +    -
                    O10     401     +    -
     (key  +  atom present for this chain 
           -  atom absent  for this chain)
 transforms for each of the superpositions orthogonal matrix r11 r12 r13 r21 r22 r23 r31 r32 r33 tx ty tz (PDBSET syntax): 
TRANS_AB -0.872754 -0.487493  0.025513 -0.487434  0.873114  0.008926 -0.026628 -0.004646 -0.999635    44.096   -12.853    15.142
 reverse transforms 
TRANS_BA -0.872754 -0.487434 -0.026628 -0.487493  0.873114 -0.004646  0.025513  0.008926 -0.999635    32.623    32.789    14.127
 
 Setting up LSSR interatomic distance restraints
 Interpreting card: NOTE BUSTER_SIM_RESTRAIN_LSSR ncsautoAB 6.666
        setting up interatomic distance pair restraints with:
        Dmin=         0.00
        Dmax=         5.50
        sigma=        0.20
        Vmax=         3.00
        weight=       6.67
        number of distance pairs defined for this set is      35359
 Have not supplied either '-sim_swap_equiv' or '-sim_swap_equiv_plus' argument 
   so will not try swaping equivalent atoms to improve NCS and/or target agreement.
 
 NCS by LSSR outlier analysis
 -autoncs option automatic NCS prune routine -------- 
 Residues pruned out by gradient condition:
       
 Residues pruned out by function/max condition:
           A|34     A|149     A|150     A|158     A|168     A|169     A|170 
          A|171     A|172     A|173     A|187     A|188     A|216     A|220 
          A|229     A|259     A|260     A|264     A|307     A|308     A|326 
          A|346    A|ENDA      B|34     B|149     B|150     B|158     B|168 
          B|169     B|170     B|171     B|172     B|173     B|187     B|188 
          B|216     B|220     B|229     B|259     B|260     B|264     B|307 
          B|308     B|326     B|346    B|ENDA 
  
 Total number of residues for which LSSR restraints have been pruned out is       46 out of      698 participating
 Number of LSSR restraints pruned out is      2850 this is    8.1% of the total           35359
  
 -autoncs .Gelly cards suitable for adaptation for manual definition/pruning
           (note auto pruning is done in a slightly different way so exact results may differ slightly)
 -autoncs option .Gelly cards also written to file  auto_gelly_cards.txt
NOTE BUSTER_SET ncsautoXcld = Water    # card added - produced by -autoncs
NOTE BUSTER_SET ncsautoXcld = ncsautoXcld + { A|34 A|149 A|150 A|158 A|168 }    # card added - produced by -autoncs
NOTE BUSTER_SET ncsautoXcld = ncsautoXcld + { A|169 A|170 A|171 A|172 A|173 }    # card added - produced by -autoncs
NOTE BUSTER_SET ncsautoXcld = ncsautoXcld + { A|187 A|188 A|216 A|220 A|229 }    # card added - produced by -autoncs
NOTE BUSTER_SET ncsautoXcld = ncsautoXcld + { A|259 A|260 A|264 A|307 A|308 }    # card added - produced by -autoncs
NOTE BUSTER_SET ncsautoXcld = ncsautoXcld + { A|326 A|346 A|ENDA }    # card added - produced by -autoncs
NOTE BUSTER_SET ncsautoABset = Chain_A \ ncsautoXcld    # card added - produced by -autoncs
NOTE BUSTER_SIM_DEFINE ncsautoAB ncsautoABset B    # card added - produced by -autoncs
NOTE BUSTER_SIM_RESTRAIN_LSSR ncsautoAB 6.666    # card added - produced by -autoncs

 LSSR function contribution before pruning    172954.1
                             after pruning    141691.7
 Graph of which residues pruned from LSSR: ncs_pruned.mtv
 Graph of LSSR function outliers ncs_function_outliers.mtv
 Graph of LSSR gradient outliers ncs_gradient_outliers.mtv
 LSSR - analysis of distance difference distribution for NCS
         DeltaD less than 0.5 Angs:  proportion of population= 95.68% average DeltaD of subset     0.123 Angs
 Histogram of LSSR deltaD distribution ncs_histogram.mtv
 
 Interpreting card: NOTE BUSTER_TLS_SET tls1 { A|* }
 Interpreting card: NOTE BUSTER_TLS_SET tls2 { B|* }
 Interpreting card: NOTE BUSTER_TLS_SET tls1 { A|* }
 Interpreting card: NOTE BUSTER_TLS_T   tls1     -1.24008E-01 -8.75701E-03  1.95905E-01 -2.54756E-03 -1.97912E-02  4.23354E-03
 Interpreting card: NOTE BUSTER_TLS_L   tls1      6.25524E-01  9.73647E-01  4.40986E-01 -4.86570E-02  4.82699E-01 -7.20310E-01
 Interpreting card: NOTE BUSTER_TLS_S   tls1      1.09103E-01 -2.36495E-01  1.88194E-01  1.15882E-01 -1.08713E-01 -1.46902E-01  1.66685E-01  4.93260E-02
 Interpreting card: NOTE BUSTER_TLS_O   tls1      2.31588E+01 -6.71772E+01  1.06797E+01
 Interpreting card: NOTE BUSTER_TLS_SET tls2 { B|* }
 Interpreting card: NOTE BUSTER_TLS_T   tls2     -1.58512E-01 -6.11579E-02  2.48458E-01  1.04689E-02 -3.47117E-02  9.11453E-03
 Interpreting card: NOTE BUSTER_TLS_L   tls2      4.70351E-01  3.82030E-01  1.72027E-01  2.16840E-01  2.63951E-01  2.62161E-01
 Interpreting card: NOTE BUSTER_TLS_S   tls2      2.83079E-02  6.71331E-03 -8.95201E-02 -3.17234E-02 -6.69705E-02  1.54405E-02  6.85666E-02 -3.05844E-02
 Interpreting card: NOTE BUSTER_TLS_O   tls2     -2.22253E+01 -7.89989E+01  3.21640E+00
 Interpreting card: NOTE BUSTER_TLS_FIX tls1 ALL
 Interpreting card: NOTE BUSTER_TLS_FIX tls2 ALL


 TLS information. Total number of groups:     2


 Group   Natoms    FixRB   FixTLS   Group Tag    Group Spec
     1     2768      Y        Y     tls1    { A|* }
     2     2758      Y        Y     tls2    { B|* }
 
NOTE BUSTER_TLS_SET tls1 { A|* }
NOTE BUSTER_TLS_T   tls1     -1.24008E-01 -8.75701E-03  1.95905E-01 -2.54756E-03 -1.97912E-02  4.23354E-03
NOTE BUSTER_TLS_L   tls1      6.25524E-01  9.73647E-01  4.40986E-01 -4.86570E-02  4.82699E-01 -7.20310E-01
NOTE BUSTER_TLS_S   tls1      1.09103E-01 -2.36495E-01  1.88194E-01  1.15882E-01 -1.08713E-01 -1.46902E-01  1.66685E-01  4.93260E-02
NOTE BUSTER_TLS_O   tls1      2.31588E+01 -6.71772E+01  1.06797E+01
NOTE BUSTER_TLS_FIX tls1 ALL
 
NOTE BUSTER_TLS_SET tls2 { B|* }
NOTE BUSTER_TLS_T   tls2     -1.58512E-01 -6.11579E-02  2.48458E-01  1.04689E-02 -3.47117E-02  9.11453E-03
NOTE BUSTER_TLS_L   tls2      4.70351E-01  3.82030E-01  1.72027E-01  2.16840E-01  2.63951E-01  2.62161E-01
NOTE BUSTER_TLS_S   tls2      2.83079E-02  6.71331E-03 -8.95201E-02 -3.17234E-02 -6.69705E-02  1.54405E-02  6.85666E-02 -3.05844E-02
NOTE BUSTER_TLS_O   tls2     -2.22253E+01 -7.89989E+01  3.21640E+00
NOTE BUSTER_TLS_FIX tls2 ALL


 TLS: mmCIF group specification begin


#
loop_
_pdbx_refine_tls_group.id
_pdbx_refine_tls_group.refine_tls_id
_pdbx_refine_tls_group.beg_PDB_ins_code
_pdbx_refine_tls_group.beg_auth_asym_id
_pdbx_refine_tls_group.beg_auth_seq_id
_pdbx_refine_tls_group.end_PDB_ins_code
_pdbx_refine_tls_group.end_auth_asym_id
_pdbx_refine_tls_group.end_auth_seq_id
_pdbx_refine_tls_group.selection
_pdbx_refine_tls_group.selection_details
   1   1 . A   -1 . A  346 . '{ A|* }'
   2   1 . A  401 . A  401 . '{ A|* }'
   3   2 . B   -1 . B  346 . '{ B|* }'
#


 TLS: mmCIF group specification end


 Interpreting card: NOTE BUSTER_ANISO_SET Empty
 Interpreting card: NOTE BUSTER_ANISO_WEIGHT SCA  16.0
 Interpreting card: NOTE BUSTER_ANISO_WEIGHT SPH   0.6    0.6
 Interpreting card: NOTE BUSTER_ANISO_WEIGHT SIM   7.8    4.8    2.6    0
 Interpreting card: NOTE BUSTER_ANISO_WEIGHT RIG  21.5   21.5    3.7    0


 After TLS and ANISO: Number of atoms with fixed/combined xyz =     0
 After TLS and ANISO: Number of atoms with fixed/combined B   =     0
 After TLS and ANISO: Number of atoms with fixed/combined OCC =  5526
 After TLS and ANISO: Number of atoms with fixed/combined BIJ =  5526
 After TLS and ANISO: Number of atoms in a TLS group          =  5526
                                     cf total number of atoms =  5526
 

 ############### Setup state vector

 No free_vect_ulim_xyz argument found: using default of  100.000
 No free_vect_llim_xyz argument found: using default of -100.000
 Number of parameters added for TLS description                 =       0
 Number of parameters to describe molecular conformation        =   22104

 ############### Setup free vector

 Number of free parameters to be adjusted in refinement   =   22104

 ############### Setting up own geometry


 No command line argument '-tnt_fn' so will use gelly to do geometry function calculations

 ============= Call TNT functions


 ============= Get TNT symmetry information

 
      Getting symmetry operators from TNT. 
          gelly will classify symmetry using pdb-like convention: 
               SYMOP   SYMMETRY
              NNNMMM   OPERATOR
                1555   X,Y,Z
                2555   -Y,X-Y,Z
                3555   -X+Y,-X,Z
                4555   -X,-Y,1/2+Z
                5555   Y,-X+Y,1/2+Z
                6555   X-Y,X,1/2+Z
          where NNN -> operator number and MMM -> translation vector
 

 ============= Own Gelly geometry/restraints setup


 ----------- Identify bond-types


    Setting up GEOMBONDREC_TYPE_HASH* to be able to map a given bond type (e.g.
    "single") to an internal integer (GBOND_TYPE=1) etc.

    Using default  GEOMBONDREC_TYPE_HASH_STR='SING* 1 DOUB* 2 TRIP* 3 AROM* 4 DELO* 4 METAL* 5':
    to override default use a NOTE BUSTER_GEOMBONDREC_TYPE_HASH_STR gelly card.

 ----------- Classify torsion into peptide omega and other


 ----------- Setup of non-bonded terms

 Ideal-distance contact term setup:
    Restraint function constants (normally these values are from NOTE BUSTER_IDEAL_CONTACT_* cards)
    Sigma for general contacts      0.200 Angs
    Sigma for 1-4 contacts          0.100 Angs (contacts across a torsion)
    Sigma for 1-5 contacts          0.150 Angs
    Sigma for 1-N contacts          0.200 Angs
    Sigma for HBOND pairs           0.100 Angs (contacts between HB_types A and D or B's)
    Contact distance increment for each atom in HBOND          -0.150 Angs 
    Contact distance increment for H-bond acceptor              0.100 Angs 
    Contact distance increment for 1-4 with an O atom          -0.050 Angs 
    Contact distance increment for 1-4 with an N atom          -0.180 Angs 
    Contact distance increment for 1-4 with an C atom          -0.370 Angs 
    Contact distance increment for 1-4 with an S atom          -0.150 Angs 
    Contact distance increment for 1-4 with an P atom          -0.150 Angs 
    Contact distance increment for 1-4 with any other atom     -0.150 Angs 
    All atoms matched for CCP4 atom type.
    Number of CCP4 atom types used is         22 (H-bound and not H-bound counted separately)

 Setting up short/ideal-distance contacts
   Using gelly special position handling. (For TNT instead rerun with argument '-special_tnt')

   No command line argument '-special_all' specified. We will auto-generate
   EXCLUDE cards only for atom-atom contacts across symmetry (i.e. on special
   position) when this involves a contact within a residue to itself.

   Have set up non-bonded exclusions - 1-3 contacts
   Atom A|27:CA         makes the most 1-3 contacts:  6

   Have set up non-bonded exclusions - 1-4 contacts
   Atom A|27:N          makes the most 1-4 contacts:  7

   Have set up non-bonded exclusions - 1-5 contacts
   Atom A|28:C.A        makes the most 1-5 contacts: 10

 ############################################################################################################
 ###################################################### setting up contact terms
 ############################################################################################################


 ============= Call Gelly versions of TNT functions

 WARNING: 1-4 contact without 1-(2,3)-4 data for A|401:C01(LIG)       and A|401:C08(LIG)       X-X
 WARNING: 1-4 contact without 1-(2,3)-4 data for A|401:C02(LIG)       and A|401:C05(LIG)       X-X
 WARNING: 1-4 contact without 1-(2,3)-4 data for A|401:C02(LIG)       and A|401:C09(LIG)       X-X
 WARNING: 1-4 contact without 1-(2,3)-4 data for A|401:C03(LIG)       and A|401:C06(LIG)       X-X
 WARNING: 1-4 contact without 1-(2,3)-4 data for A|401:N04(LIG)       and A|401:N07(LIG)       X-X
 WARNING: 1-4 contact without 1-(2,3)-4 data for A|401:C05(LIG)       and A|401:C08(LIG)       X-X

       TERM      weight      number      TNT_fn    Gelly_fn        diff
       BOND       2.000        5642    5582.638    5582.638       0.000
       ANGL       2.000        7656   12526.384   12526.384       0.000
       IMPR       0.000           0       0.000       0.000       0.000
       TORS       2.000        1964    3021.592    3021.592      -0.000
       TRIG       2.000           0       0.000       0.000       0.000
       PLAN       5.000         958   15357.010   15357.010       0.000
       CONT       5.000           0    2062.178       0.000    2062.178
       BCOR      10.000        5632  273215.507  276337.536   -3122.029


 ============= Call Gelly geometry functions

   Switching torsion functional form to sinusoidal rather than split harmonic functional.
      if you want this not to happen then rerun with '-torsharm'
      Weight for sinusoidal torsion=     2.000
   BCOR function/gradient will be split between H and non-H atoms (as for any atom pair). Use '-bcorrel_all_in_hydrogens' to change this.

 ----------- Fix B-correlation restraints

 
 Checking for bonds that do not have a bcorrel restraint defined
 We will wipe all existing BCORrels if OCC=0.0 atom(s) is/are involved. This can be' switched off via the '-bcorrel_nowipe_zeroocc' flag.
 We will wipe all existing BCORrels if only non-hydrogen atom(s) with fixed B(iso) are involved. This can be switched off via the '-bcorrel_nowipe_fixb_nonhydrogens' flag.
 We will keep all existing BCORrels if hydrogen atom(s) with fixed B(iso) is/are involved. This can be switched off via the '-bcorrel_wipe_fixb_hydrogens' flag.
 No -bcorrel_missing_sigma found. So will set sigma to     2.5 Ang**2 for any bond with no existing BCORrel
 No -bcorrel_missing_sigma_hydrogens found. So will set sigma to     1.0 Ang**2 for any bond with no existing BCORrel involving hydrogens

 No active BCORels found involving OCC=0.0 atom(s)


 No active BCORels found involving fixed-B (only) non-hydrogen atom(s)

    add a default BCOR for bond A|401:C01(LIG) A|401:C02(LIG)
    add a default BCOR for bond A|401:C02(LIG) A|401:C03(LIG)
    add a default BCOR for bond A|401:C02(LIG) A|401:N07(LIG)
    add a default BCOR for bond A|401:C03(LIG) A|401:N04(LIG)
    add a default BCOR for bond A|401:N04(LIG) A|401:C05(LIG)
    add a default BCOR for bond A|401:C05(LIG) A|401:C06(LIG)
    add a default BCOR for bond A|401:C06(LIG) A|401:N07(LIG)
    add a default BCOR for bond A|401:N07(LIG) A|401:C08(LIG)
    add a default BCOR for bond A|401:C08(LIG) A|401:C09(LIG)
    add a default BCOR for bond A|401:C09(LIG) A|401:O10(LIG)

 Have added a total of        10 BCORrels for bonds which do not already have a BCORrel


 No bonds (for potential BCORrel addition) were skipped due to both atoms having constant/fixed B(iso)


 ----------- Deactivate hydrogen-free planes if hydrogen atoms present

 A total of       0 planes have been deactivated because they are duplicated hydrogen-free and with-hydrogen forms.

 ----------- Handle peptide omega angle(s)

      Torsion angles split into peptide omega and other
         Number of peptide omega angles is       698
         Number of other torsion is             1266
      Peptide omega angles are monitored rather than directly restrained (PLANE used instead).

 ----------- Fix cis-PRO

 Now apply the fix to get correct restraints for cis PRO residues
   Have got      11 bonds records and      26 angles
   from GEOMETRY cards for the special PROC residue
   Weight for ideal-distance contact term=     4.000

 ----------- Prepare SSBOND/LINK for output PDB file


 ----------- Prepare for QM


 ----------- Prepare symmetry in utility distance restraints


 ############### Setting up Rhofit contacts (if needed)

 No ligand-(fixed protein) contact term will be used

 ############### Working out initial function and gradient


   MaxLik Scaling Cycle 0002
 
 Initial functional value = 6.038013E+05 rms gradient=      986.652
 X-ray contribution to function value = 1.248625E+05
 Geometry contribution (inc ncs)      = 4.789388E+05
 Geometry function summary
     term                           number     weight       rms        GooF      function     fn/numb
     ================================================================================================
     BOND bond lengths (angs)       5642       2.000      0.0141       0.703      5582.64       0.989
     ANGL bond angles (degs)        7656       2.000        1.52       0.904     12526.38       1.636
     TORS split-harmonic tors       1266       0.000       17.36       1.092         0.00       0.000
     SINTOR sinusoidal tors         1266       2.000       17.36                  2343.77       1.851
     PLAN general plane (angs)       958       5.000      0.0358       1.791     15357.01      16.030
     BCORrelations (angs^2)         5642      10.000       6.203       2.213    276345.13      48.980
     CONT (bad contacts)               0       5.000                                 0.00
     IDEAL(ideal-dist contact)      3902       4.000                             25062.19
     CHIRAL (gelly semiharmon)       742       5.000                                29.99
     SIM similarity restraint for NCS and/or TARGET                             141691.69
     OCCSUM restraint on sum of occupanies                                           0.00       as none defined
     DISTAN utility restraints interatomic distance                                  0.00       as none defined
     UTILANGLE utility angle restraints                                              0.00       as none defined
     UTILTOR utility restraints on torsion angles                                    0.00       as none defined
     Peptide omega torsion angles                           7.79 degs - monitor only
 
 WARNING: Have |delta/sigma| deviations > 5.0 sigma. Number of outliers for each term: 
 WARNING:         7 bond angles.  Worst is     7.4 sigs    95.52 degs A|28:CB.A=CA.A=C.A (ASN)
 WARNING:        14 planes.       Worst is     7.3 sigs     0.15 Angs A|27:C=A|27:CA=A|27:O=A|28:N.B=A|28:CA.B
 WARNING:        12 idealD contacts. Worst    13.2 sigs     1.68 Angs A|285:CB(THR)=A|401:C06(LIG) symm: 1555=3655
 WARNING:       223 Bcorrel.      Worst is    14.2 sigs   -38.15 Angs^2 A|248:CA=C (LEU)

 Weighted rms      0.0141         1.517  (temporary rms bond and angle for BUSTER to pick up!)
 Weighted intra-residual rms(bond) for StdProtein (excluding H)      0.0146

 ############### Running initial check


 Initial Geometry Sanity Check

 Check for duplicate bonds: no duplicates found
 
 Check for duplicate or missing bond angles: all are OK
 Check for bond between an atom and itself not involving symmetry:  all are OK
 *** WARNING: There are 5 bad contacts where Delta/sigma is greater than      6.5
 *** WARNING: Looking at each one in turn to see whether it is likely to indicate a setup problem with geometry restraints.

        1 dist=   1.68 idealD=   3.00 sigma=   0.10 abs(Delta/sigma)=   13.21
          Between atoms   A|285:CB (THR)          and   A|401:C06 (LIG)         (C-C)
          occupancies=     1.00        0.78
          The contact is between two atoms via a symmetry operator.
 [check-012]
          One atom has occupancy below 1. Hopefully refinement will fix this.

        2 dist=   2.27 idealD=   2.75 sigma=   0.04 abs(Delta/sigma)=   11.19
          Between atoms   A|28:CB.A (ASN)         and   A|28:O.A (ASN)          (C-O)
          (both are in the same alternate conformation)
          occupancies=     0.41        0.41
 [check-033]
          This is a contact within a residue - so probably just a distorted
          residue?

        3 dist=   2.39 idealD=   2.75 sigma=   0.04 abs(Delta/sigma)=    8.47
          Between atoms   B|28:CB.A (ASN)         and   B|28:O.A (ASN)          (C-O)
          (both are in the same alternate conformation)
          occupancies=     0.41        0.41
 [check-033]
          This is a contact within a residue - so probably just a distorted
          residue?

        4 dist=   2.32 idealD=   3.33 sigma=   0.14 abs(Delta/sigma)=    7.33
          Between atoms   A|285:O (THR)           and   A|401:C02 (LIG)         (O-C)
          occupancies=     1.00        0.78
          The contact is between two atoms via a symmetry operator.
 [check-012]
          One atom has occupancy below 1. Hopefully refinement will fix this.

        5 dist=   2.09 idealD=   3.49 sigma=   0.21 abs(Delta/sigma)=    6.54
          Between atoms   A|285:OG1 (THR)         and   A|401:C06 (LIG)         (O-C)
          occupancies=     1.00        0.78
          The contact is between two atoms via a symmetry operator.
 [check-012]
          One atom has occupancy below 1. Hopefully refinement will fix this.
 GEOMETRY CHECK OVERALL:  WARN


 ############### Creating geometry/screen report


 ############### Option "tao" - run Tao optimizer

 TAO: Toolkit for Advanced Optimization. Reference: 
     Munson, T., Sarich, J., Wild, S., Benson, S., McInnes, L.C. (2018)
     "TAO User Manual (Revision 3.9)", Mathematics and Computer Science
     Division, Argonne National Laboratory
     ANL/MCS-TM-322, http://www.mcs.anl.gov/petsc/
 
 Have found -keeppdb flag so will write out a .pdb file every 1000000 its during opt
 Will apply TAO optimization 
 Maximum number of iterations      100
 Convergence test on Grms       4.0000 (-glim)
 Limit on rms displacement to original position (rmsD2init): NONE test inactive (-dlim)

    analysing argument no 0 : -autoncs with j=0
    analysing argument no 1 : -hydrogens_input_occ2zero with j=0
    analysing argument no 2 : -hydrogens_output_occ2heavy with j=0
    analysing argument no 3 : -screen with j=0
    analysing argument no 4 : 100 with j=0
    analysing argument no 5 : -screen_sigma with j=0
    analysing argument no 6 : 3.0 with j=0
    analysing argument no 7 : -keeppdb with j=0
    analysing argument no 8 : 1000000 with j=0
    analysing argument no 9 : -taomonitor_verbose with j=0
    found argument starting with "-tao" : -taomonitor_verbose
    analysing argument no 10 : 2 with j=1
    found value after -tao/-options_/-viewer_ argument : "2"
 PETSC/TAO: setting option "-taomonitor_verbose" = "2"


   MaxLik Scaling Cycle 0003
   Iters     Tot_Funct    Xray_Funct    Geom_Funct          Grms     rmsD2init     maxD2init             R         Rfree           LLG       LLGfree     rms(bond)    rms(angle)       Opttag_H
   Iters             K   B_IMPF_FRAG        K_MISS   B_TEMP_MISS   K_IMPF_MISS   B_IMPF_MISS        K_SOLV   B_TEMP_SOLV   K_IMPF_SOLV   B_IMPF_SOLV        B_TEMP     B_TEMP_11     B_TEMP_12     B_TEMP_22     B_TEMP_13     B_TEMP_23     B_TEMP_33      K_WILSON      B_WILSON      FGCYCLES       Scatag_H
   Iters          BOND         ANGLE      IMPROPER       TORSION    SINTORSION      TRIGONAL        PLANAR  BCORRELATION       CONTACT     IDEALDIST        CHIRAL    SIMILARITY       OCCSSUM  UTILDISTANCE     UTILANGLE   UTILTORSION        RHOFIT        ADP-01        ADP-02        ADP-03        ADP-04        ADP-05        ADP-06        ADP-07        ADP-08        ADP-09        ADP-10      RHOFITPC            QM       Funtag_H
       0  6.036859E+05  1.248625E+05  4.788234E+05       983.369  0.000000E+00  0.000000E+00      0.225379      0.218586  0.000000E+00  0.000000E+00     0.0141312       1.51712       Opttag_D
       0       61.1303       4.09166       1.00000  0.000000E+00       1.00000  0.000000E+00      0.909842       62.1835       1.00000       10.7663  0.000000E+00      0.719578  0.000000E+00      0.719578  0.000000E+00  0.000000E+00      -1.43916       58.0434     -0.634584        2       Scatag_D
       0       5582.64       12526.4  0.000000E+00  0.000000E+00       2343.77  0.000000E+00       15357.0  2.762297E+05  0.000000E+00       25062.2       29.9854  1.416917E+05 -1.000000E+10 -1.000000E+10 -1.000000E+10 -1.000000E+10 -1.000000E+10  0.000000E+00  0.000000E+00  0.000000E+00  0.000000E+00  0.000000E+00  0.000000E+00  0.000000E+00  0.000000E+00  0.000000E+00  0.000000E+00  0.000000E+00  0.000000E+00       Funtag_D
   Geometry Restraint Deviations SCREEN Information

 

   MaxLik Scaling Cycle 0004

   MaxLik Scaling Cycle 0005

   Wilson plot

   Statistics in resolution bins after refinement cycle 0001
```


|  |  |
| --- | --- |
|  | - Rxpct factors plot- Rxpct factors table- <Log-Lik Gain>- Ln Sigma A- Correlation Coefficients |


```
   Iters     Tot_Funct    Xray_Funct    Geom_Funct          Grms     rmsD2init     maxD2init             R         Rfree           LLG       LLGfree     rms(bond)    rms(angle)
   Iters             K   B_IMPF_FRAG        K_MISS   B_TEMP_MISS   K_IMPF_MISS   B_IMPF_MISS        K_SOLV   B_TEMP_SOLV   K_IMPF_SOLV   B_IMPF_SOLV        B_TEMP     B_TEMP_11     B_TEMP_12     B_TEMP_22     B_TEMP_13     B_TEMP_23     B_TEMP_33      K_WILSON      B_WILSON      FGCYCLES
   Iters          BOND         ANGLE      IMPROPER       TORSION    SINTORSION      TRIGONAL        PLANAR  BCORRELATION       CONTACT     IDEALDIST        CHIRAL    SIMILARITY       OCCSSUM  UTILDISTANCE     UTILANGLE   UTILTORSION        RHOFIT        ADP-01        ADP-02        ADP-03        ADP-04        ADP-05        ADP-06        ADP-07        ADP-08        ADP-09        ADP-10      RHOFITPC            QM
       1  4.646435E+05  1.257003E+05  3.389432E+05       617.702    0.00725627     0.0399728      0.226896      0.220314   -0.00722158   -0.00605894     0.0108412       1.36820       Opttag_D
       1       60.9955       4.22244       1.00000  0.000000E+00       1.00000  0.000000E+00      0.911549       62.3372       1.00000       10.2583  0.000000E+00      0.938019  0.000000E+00      0.938019  0.000000E+00  0.000000E+00      -1.87604       58.0434     -0.634584        2       Scatag_D
       1       3285.72       10187.9  0.000000E+00  0.000000E+00       2334.69  0.000000E+00       13392.8  1.544008E+05  0.000000E+00       22984.3       20.2894  1.323366E+05 -1.000000E+10 -1.000000E+10 -1.000000E+10 -1.000000E+10 -1.000000E+10  0.000000E+00  0.000000E+00  0.000000E+00  0.000000E+00  0.000000E+00  0.000000E+00  0.000000E+00  0.000000E+00  0.000000E+00  0.000000E+00  0.000000E+00  0.000000E+00       Funtag_D
   Geometry Restraint Deviations SCREEN Information

 

   MaxLik Scaling Cycle 0006

   Wilson plot

   Statistics in resolution bins after refinement cycle 0002
```


|  |  |
| --- | --- |
|  | - Rxpct factors plot- Rxpct factors table- <Log-Lik Gain>- Ln Sigma A- Correlation Coefficients |


```
   Iters     Tot_Funct    Xray_Funct    Geom_Funct          Grms     rmsD2init     maxD2init             R         Rfree           LLG       LLGfree     rms(bond)    rms(angle)
   Iters             K   B_IMPF_FRAG        K_MISS   B_TEMP_MISS   K_IMPF_MISS   B_IMPF_MISS        K_SOLV   B_TEMP_SOLV   K_IMPF_SOLV   B_IMPF_SOLV        B_TEMP     B_TEMP_11     B_TEMP_12     B_TEMP_22     B_TEMP_13     B_TEMP_23     B_TEMP_33      K_WILSON      B_WILSON      FGCYCLES
   Iters          BOND         ANGLE      IMPROPER       TORSION    SINTORSION      TRIGONAL        PLANAR  BCORRELATION       CONTACT     IDEALDIST        CHIRAL    SIMILARITY       OCCSSUM  UTILDISTANCE     UTILANGLE   UTILTORSION        RHOFIT        ADP-01        ADP-02        ADP-03        ADP-04        ADP-05        ADP-06        ADP-07        ADP-08        ADP-09        ADP-10      RHOFITPC            QM
       2  4.073671E+05  1.260016E+05  2.813655E+05       411.959     0.0129846     0.0719509      0.227590      0.221162   -0.00981864   -0.00811949    0.00964311       1.30401       Opttag_D
       2       60.9190       4.26463       1.00000  0.000000E+00       1.00000  0.000000E+00      0.911800       62.3248       1.00000       10.1374  0.000000E+00       1.03905  0.000000E+00       1.03905  0.000000E+00  0.000000E+00      -2.07809       58.0434     -0.634584        1       Scatag_D
       2       2599.64       9254.37  0.000000E+00  0.000000E+00       2325.85  0.000000E+00       11906.3  1.098933E+05  0.000000E+00       21468.3       24.4745  1.238933E+05 -1.000000E+10 -1.000000E+10 -1.000000E+10 -1.000000E+10 -1.000000E+10  0.000000E+00  0.000000E+00  0.000000E+00  0.000000E+00  0.000000E+00  0.000000E+00  0.000000E+00  0.000000E+00  0.000000E+00  0.000000E+00  0.000000E+00  0.000000E+00       Funtag_D
   Geometry Restraint Deviations SCREEN Information

 
       3  3.614688E+05  1.266709E+05  2.347979E+05       478.789     0.0258476      0.139809      0.229171      0.222817    -0.0155877    -0.0131392     0.0122879       1.49406       Opttag_D
       3       60.8314       4.37516       1.00000  0.000000E+00       1.00000  0.000000E+00      0.912705       62.3427       1.00000       9.90920  0.000000E+00       1.22422  0.000000E+00       1.22422  0.000000E+00  0.000000E+00      -2.44843       58.0434     -0.634584        1       Scatag_D
       3       4221.19       12148.4  0.000000E+00  0.000000E+00       2311.70  0.000000E+00       9311.16       81650.5  0.000000E+00       18882.2       75.1715  1.061975E+05 -1.000000E+10 -1.000000E+10 -1.000000E+10 -1.000000E+10 -1.000000E+10  0.000000E+00  0.000000E+00  0.000000E+00  0.000000E+00  0.000000E+00  0.000000E+00  0.000000E+00  0.000000E+00  0.000000E+00  0.000000E+00  0.000000E+00  0.000000E+00       Funtag_D
       4  3.367024E+05  1.270840E+05  2.096184E+05       415.656     0.0347306      0.182859      0.230192      0.223716    -0.0191478    -0.0162781     0.0134579       1.45224       Opttag_D
       4       60.7716       4.44212       1.00000  0.000000E+00       1.00000  0.000000E+00      0.913136       62.2825       1.00000       9.81069  0.000000E+00       1.31917  0.000000E+00       1.31917  0.000000E+00  0.000000E+00      -2.63835       58.0434     -0.634584        1       Scatag_D
       4       5063.27       11477.8  0.000000E+00  0.000000E+00       2303.48  0.000000E+00       7968.84       70894.8  0.000000E+00       17542.0       128.424       94239.8 -1.000000E+10 -1.000000E+10 -1.000000E+10 -1.000000E+10 -1.000000E+10  0.000000E+00  0.000000E+00  0.000000E+00  0.000000E+00  0.000000E+00  0.000000E+00  0.000000E+00  0.000000E+00  0.000000E+00  0.000000E+00  0.000000E+00  0.000000E+00       Funtag_D
       5  3.193735E+05  1.271113E+05  1.922622E+05       305.210     0.0381988      0.195484      0.230350      0.223859    -0.0193838    -0.0163747     0.0125973       1.32685       Opttag_D
       5       60.7207       4.43394       1.00000  0.000000E+00       1.00000  0.000000E+00      0.912665       62.1541       1.00000       9.87277  0.000000E+00       1.33436  0.000000E+00       1.33436  0.000000E+00  0.000000E+00      -2.66872       58.0434     -0.634584        1       Scatag_D
       5       4436.42       9581.35  0.000000E+00  0.000000E+00       2300.44  0.000000E+00       7588.31       61810.9  0.000000E+00       17132.4       123.141       89289.2 -1.000000E+10 -1.000000E+10 -1.000000E+10 -1.000000E+10 -1.000000E+10  0.000000E+00  0.000000E+00  0.000000E+00  0.000000E+00  0.000000E+00  0.000000E+00  0.000000E+00  0.000000E+00  0.000000E+00  0.000000E+00  0.000000E+00  0.000000E+00       Funtag_D
       6  2.838301E+05  1.275586E+05  1.562716E+05       268.948     0.0555493      0.253718      0.231804      0.225389    -0.0232388    -0.0199883     0.0116928       1.33857       Opttag_D
       6       60.5517       4.47152       1.00000  0.000000E+00       1.00000  0.000000E+00      0.911559       61.7301       1.00000       10.0294  0.000000E+00       1.43434  0.000000E+00       1.43434  0.000000E+00  0.000000E+00      -2.86869       58.0434     -0.634584        1       Scatag_D
       6       3822.19       9751.42  0.000000E+00  0.000000E+00       2292.19  0.000000E+00       6214.47       47891.3  0.000000E+00       15584.9       134.333       70580.7 -1.000000E+10 -1.000000E+10 -1.000000E+10 -1.000000E+10 -1.000000E+10  0.000000E+00  0.000000E+00  0.000000E+00  0.000000E+00  0.000000E+00  0.000000E+00  0.000000E+00  0.000000E+00  0.000000E+00  0.000000E+00  0.000000E+00  0.000000E+00       Funtag_D
       7  2.781577E+05  1.279990E+05  1.501587E+05       548.871     0.0685458      0.290696      0.233132      0.226674    -0.0270352    -0.0239166     0.0132440       1.86167       Opttag_D
       7       60.4444       4.52314       1.00000  0.000000E+00       1.00000  0.000000E+00      0.911048       61.4478       1.00000       10.1110  0.000000E+00       1.51281  0.000000E+00       1.51281  0.000000E+00  0.000000E+00      -3.02561       58.0434     -0.634584        1       Scatag_D
       7       4903.59       18862.0  0.000000E+00  0.000000E+00       2288.36  0.000000E+00       5579.64       42565.4  0.000000E+00       14768.4       132.962       61058.4 -1.000000E+10 -1.000000E+10 -1.000000E+10 -1.000000E+10 -1.000000E+10  0.000000E+00  0.000000E+00  0.000000E+00  0.000000E+00  0.000000E+00  0.000000E+00  0.000000E+00  0.000000E+00  0.000000E+00  0.000000E+00  0.000000E+00  0.000000E+00       Funtag_D
       8  2.639355E+05  1.279990E+05  1.359365E+05       235.862     0.0692841      0.290797      0.233154      0.226690    -0.0270353    -0.0239509     0.0112799       1.35848       Opttag_D
       8       60.4253       4.51796       1.00000  0.000000E+00       1.00000  0.000000E+00      0.910854       61.4094       1.00000       10.1286  0.000000E+00       1.51465  0.000000E+00       1.51465  0.000000E+00  0.000000E+00      -3.02929       58.0434     -0.634584        1       Scatag_D
       8       3557.06       10043.6  0.000000E+00  0.000000E+00       2287.68  0.000000E+00       5545.19       39902.7  0.000000E+00       14685.5       117.584       59797.2 -1.000000E+10 -1.000000E+10 -1.000000E+10 -1.000000E+10 -1.000000E+10  0.000000E+00  0.000000E+00  0.000000E+00  0.000000E+00  0.000000E+00  0.000000E+00  0.000000E+00  0.000000E+00  0.000000E+00  0.000000E+00  0.000000E+00  0.000000E+00       Funtag_D
       9  2.547259E+05  1.280892E+05  1.266366E+05       163.645     0.0729228      0.296638      0.233446      0.226944    -0.0278128    -0.0248263     0.0105781       1.29483       Opttag_D
       9       60.3759       4.52163       1.00000  0.000000E+00       1.00000  0.000000E+00      0.910503       61.3066       1.00000       10.1580  0.000000E+00       1.53218  0.000000E+00       1.53218  0.000000E+00  0.000000E+00      -3.06437       58.0434     -0.634584        1       Scatag_D
       9       3128.18       9124.55  0.000000E+00  0.000000E+00       2285.76  0.000000E+00       5395.14       35744.4  0.000000E+00       14427.0       92.9746       56438.6 -1.000000E+10 -1.000000E+10 -1.000000E+10 -1.000000E+10 -1.000000E+10  0.000000E+00  0.000000E+00  0.000000E+00  0.000000E+00  0.000000E+00  0.000000E+00  0.000000E+00  0.000000E+00  0.000000E+00  0.000000E+00  0.000000E+00  0.000000E+00       Funtag_D
      10  2.467485E+05  1.282768E+05  1.184717E+05       199.611     0.0793569      0.323706      0.234033      0.227415    -0.0294290    -0.0265042     0.0102569       1.31560       Opttag_D
      10       60.3072       4.53839       1.00000  0.000000E+00       1.00000  0.000000E+00      0.910065       61.1533       1.00000       10.1995  0.000000E+00       1.56225  0.000000E+00       1.56225  0.000000E+00  0.000000E+00      -3.12450       58.0434     -0.634584        1       Scatag_D
      10       2941.12       9419.56  0.000000E+00  0.000000E+00       2283.35  0.000000E+00       5150.49       32757.5  0.000000E+00       14032.2       75.6067       51812.0 -1.000000E+10 -1.000000E+10 -1.000000E+10 -1.000000E+10 -1.000000E+10  0.000000E+00  0.000000E+00  0.000000E+00  0.000000E+00  0.000000E+00  0.000000E+00  0.000000E+00  0.000000E+00  0.000000E+00  0.000000E+00  0.000000E+00  0.000000E+00       Funtag_D
      11  2.341984E+05  1.285072E+05  1.056912E+05       233.383     0.0891951      0.378839      0.234822      0.227932    -0.0314155    -0.0282812    0.00971635       1.33097       Opttag_D
      11       60.1979       4.55337       1.00000  0.000000E+00       1.00000  0.000000E+00      0.909182       60.9113       1.00000       10.2818  0.000000E+00       1.59335  0.000000E+00       1.59335  0.000000E+00  0.000000E+00      -3.18670       58.0434     -0.634584        1       Scatag_D
      11       2639.28       9641.00  0.000000E+00  0.000000E+00       2277.49  0.000000E+00       4775.41       27874.5  0.000000E+00       13449.3       66.5711       44967.6 -1.000000E+10 -1.000000E+10 -1.000000E+10 -1.000000E+10 -1.000000E+10  0.000000E+00  0.000000E+00  0.000000E+00  0.000000E+00  0.000000E+00  0.000000E+00  0.000000E+00  0.000000E+00  0.000000E+00  0.000000E+00  0.000000E+00  0.000000E+00       Funtag_D
      12  2.284608E+05  1.287414E+05       99719.4       272.087     0.0971265      0.427085      0.235617      0.228573    -0.0334339    -0.0301839    0.00983266       1.36806       Opttag_D
      12       60.1247       4.57784       1.00000  0.000000E+00       1.00000  0.000000E+00      0.908625       60.7383       1.00000       10.3455  0.000000E+00       1.62456  0.000000E+00       1.62456  0.000000E+00  0.000000E+00      -3.24913       58.0434     -0.634584        2       Scatag_D
      12       2702.84       10185.8  0.000000E+00  0.000000E+00       2275.21  0.000000E+00       4517.43       25439.1  0.000000E+00       13063.3       66.9877       41468.7 -1.000000E+10 -1.000000E+10 -1.000000E+10 -1.000000E+10 -1.000000E+10  0.000000E+00  0.000000E+00  0.000000E+00  0.000000E+00  0.000000E+00  0.000000E+00  0.000000E+00  0.000000E+00  0.000000E+00  0.000000E+00  0.000000E+00  0.000000E+00       Funtag_D
      13  2.210367E+05  1.288763E+05       92160.4       143.036      0.102501      0.472608      0.236127      0.228897    -0.0345969    -0.0311044    0.00927108       1.21581       Opttag_D
      13       60.0724       4.58965       1.00000  0.000000E+00       1.00000  0.000000E+00      0.908112       60.6176       1.00000       10.3975  0.000000E+00       1.64126  0.000000E+00       1.64126  0.000000E+00  0.000000E+00      -3.28251       58.0434     -0.634584        1       Scatag_D
      13       2402.92       8044.84  0.000000E+00  0.000000E+00       2271.71  0.000000E+00       4344.28       23179.5  0.000000E+00       12806.4       69.0597       39041.6 -1.000000E+10 -1.000000E+10 -1.000000E+10 -1.000000E+10 -1.000000E+10  0.000000E+00  0.000000E+00  0.000000E+00  0.000000E+00  0.000000E+00  0.000000E+00  0.000000E+00  0.000000E+00  0.000000E+00  0.000000E+00  0.000000E+00  0.000000E+00       Funtag_D
      14  2.152821E+05  1.289582E+05       86323.9       124.026      0.106095      0.503478      0.236501      0.229118    -0.0353025    -0.0314666    0.00903405       1.18923       Opttag_D
      14       60.0255       4.59337       1.00000  0.000000E+00       1.00000  0.000000E+00      0.907591       60.5225       1.00000       10.4396  0.000000E+00       1.65598  0.000000E+00       1.65598  0.000000E+00  0.000000E+00      -3.31197       58.0434     -0.634584        1       Scatag_D
      14       2281.62       7696.88  0.000000E+00  0.000000E+00       2267.19  0.000000E+00       4149.17       20713.3  0.000000E+00       12570.5       71.3701       36573.8 -1.000000E+10 -1.000000E+10 -1.000000E+10 -1.000000E+10 -1.000000E+10  0.000000E+00  0.000000E+00  0.000000E+00  0.000000E+00  0.000000E+00  0.000000E+00  0.000000E+00  0.000000E+00  0.000000E+00  0.000000E+00  0.000000E+00  0.000000E+00       Funtag_D
      15  2.108609E+05  1.290878E+05       81773.1       164.069      0.110351      0.535473      0.237042      0.229544    -0.0364201    -0.0322863    0.00905828       1.20128       Opttag_D
      15       59.9680       4.60356       1.00000  0.000000E+00       1.00000  0.000000E+00      0.906985       60.4120       1.00000       10.4835  0.000000E+00       1.68044  0.000000E+00       1.68044  0.000000E+00  0.000000E+00      -3.36088       58.0434     -0.634584        1       Scatag_D
      15       2293.88       7853.60  0.000000E+00  0.000000E+00       2262.82  0.000000E+00       3915.12       19138.9  0.000000E+00       12290.9       74.9827       33942.9 -1.000000E+10 -1.000000E+10 -1.000000E+10 -1.000000E+10 -1.000000E+10  0.000000E+00  0.000000E+00  0.000000E+00  0.000000E+00  0.000000E+00  0.000000E+00  0.000000E+00  0.000000E+00  0.000000E+00  0.000000E+00  0.000000E+00  0.000000E+00       Funtag_D
      16  2.063763E+05  1.292196E+05       77156.7       137.571      0.113685      0.573895      0.237556      0.229952    -0.0375561    -0.0331709    0.00882920       1.16565       Opttag_D
      16       59.9136       4.61552       1.00000  0.000000E+00       1.00000  0.000000E+00      0.906478       60.3204       1.00000       10.5058  0.000000E+00       1.70872  0.000000E+00       1.70872  0.000000E+00  0.000000E+00      -3.41744       58.0434     -0.634584        1       Scatag_D
      16       2179.32       7394.70  0.000000E+00  0.000000E+00       2258.80  0.000000E+00       3699.76       17881.0  0.000000E+00       12030.3       77.0360       31635.8 -1.000000E+10 -1.000000E+10 -1.000000E+10 -1.000000E+10 -1.000000E+10  0.000000E+00  0.000000E+00  0.000000E+00  0.000000E+00  0.000000E+00  0.000000E+00  0.000000E+00  0.000000E+00  0.000000E+00  0.000000E+00  0.000000E+00  0.000000E+00       Funtag_D
      17  2.024346E+05  1.293349E+05       73099.6       111.686      0.115977      0.603164      0.237967      0.230297    -0.0385500    -0.0340316    0.00855488       1.14164       Opttag_D
      17       59.8702       4.62765       1.00000  0.000000E+00       1.00000  0.000000E+00      0.906141       60.2528       1.00000       10.5095  0.000000E+00       1.73318  0.000000E+00       1.73318  0.000000E+00  0.000000E+00      -3.46636       58.0434     -0.634584        1       Scatag_D
      17       2046.00       7093.17  0.000000E+00  0.000000E+00       2255.72  0.000000E+00       3542.97       16357.9  0.000000E+00       11817.8       74.9430       29911.1 -1.000000E+10 -1.000000E+10 -1.000000E+10 -1.000000E+10 -1.000000E+10  0.000000E+00  0.000000E+00  0.000000E+00  0.000000E+00  0.000000E+00  0.000000E+00  0.000000E+00  0.000000E+00  0.000000E+00  0.000000E+00  0.000000E+00  0.000000E+00       Funtag_D
      18  1.984081E+05  1.295286E+05       68879.5       130.543      0.119953      0.650477      0.238639      0.230919    -0.0402193    -0.0356070    0.00841502       1.14860       Opttag_D
      18       59.8060       4.65028       1.00000  0.000000E+00       1.00000  0.000000E+00      0.905637       60.1457       1.00000       10.5167  0.000000E+00       1.76945  0.000000E+00       1.76945  0.000000E+00  0.000000E+00      -3.53890       58.0434     -0.634584        1       Scatag_D
      18       1979.65       7179.90  0.000000E+00  0.000000E+00       2251.88  0.000000E+00       3360.84       14701.1  0.000000E+00       11502.7       70.9096       27832.5 -1.000000E+10 -1.000000E+10 -1.000000E+10 -1.000000E+10 -1.000000E+10  0.000000E+00  0.000000E+00  0.000000E+00  0.000000E+00  0.000000E+00  0.000000E+00  0.000000E+00  0.000000E+00  0.000000E+00  0.000000E+00  0.000000E+00  0.000000E+00       Funtag_D
      19  1.955611E+05  1.296560E+05       65905.1       125.279      0.123141      0.686887      0.239100      0.231329    -0.0413170    -0.0365739    0.00829596       1.13649       Opttag_D
      19       59.7638       4.66493       1.00000  0.000000E+00       1.00000  0.000000E+00      0.905238       60.0632       1.00000       10.5345  0.000000E+00       1.79166  0.000000E+00       1.79166  0.000000E+00  0.000000E+00      -3.58331       58.0434     -0.634584        1       Scatag_D
      19       1924.03       7029.39  0.000000E+00  0.000000E+00       2248.42  0.000000E+00       3270.97       13520.6  0.000000E+00       11272.6       69.8567       26569.3 -1.000000E+10 -1.000000E+10 -1.000000E+10 -1.000000E+10 -1.000000E+10  0.000000E+00  0.000000E+00  0.000000E+00  0.000000E+00  0.000000E+00  0.000000E+00  0.000000E+00  0.000000E+00  0.000000E+00  0.000000E+00  0.000000E+00  0.000000E+00       Funtag_D
      20  1.929696E+05  1.297273E+05       63242.3       94.1730      0.125283      0.711414      0.239368      0.231539    -0.0419322    -0.0370641    0.00800088       1.11305       Opttag_D
      20       59.7392       4.67289       1.00000  0.000000E+00       1.00000  0.000000E+00      0.904969       60.0101       1.00000       10.5503  0.000000E+00       1.80296  0.000000E+00       1.80296  0.000000E+00  0.000000E+00      -3.60592       58.0434     -0.634584        1       Scatag_D
      20       1789.59       6742.32  0.000000E+00  0.000000E+00       2245.72  0.000000E+00       3220.45       12486.3  0.000000E+00       11103.1       69.5372       25585.2 -1.000000E+10 -1.000000E+10 -1.000000E+10 -1.000000E+10 -1.000000E+10  0.000000E+00  0.000000E+00  0.000000E+00  0.000000E+00  0.000000E+00  0.000000E+00  0.000000E+00  0.000000E+00  0.000000E+00  0.000000E+00  0.000000E+00  0.000000E+00       Funtag_D
      21  1.900770E+05  1.298911E+05       60185.9       104.761      0.129989      0.762877      0.239968      0.232018    -0.0433438    -0.0382512    0.00777588       1.09909       Opttag_D
      21       59.6962       4.69411       1.00000  0.000000E+00       1.00000  0.000000E+00      0.904474       59.9128       1.00000       10.5814  0.000000E+00       1.82731  0.000000E+00       1.82731  0.000000E+00  0.000000E+00      -3.65461       58.0434     -0.634584        1       Scatag_D
      21       1690.36       6574.35  0.000000E+00  0.000000E+00       2241.39  0.000000E+00       3133.38       11743.6  0.000000E+00       10785.0       69.4751       23948.5 -1.000000E+10 -1.000000E+10 -1.000000E+10 -1.000000E+10 -1.000000E+10  0.000000E+00  0.000000E+00  0.000000E+00  0.000000E+00  0.000000E+00  0.000000E+00  0.000000E+00  0.000000E+00  0.000000E+00  0.000000E+00  0.000000E+00  0.000000E+00       Funtag_D
      22  1.882702E+05  1.300519E+05       58218.3       129.796      0.134157      0.807810      0.240548      0.232508    -0.0447297    -0.0395071    0.00778425       1.11042       Opttag_D
      22       59.6610       4.71599       1.00000  0.000000E+00       1.00000  0.000000E+00      0.904045       59.8313       1.00000       10.6132  0.000000E+00       1.85221  0.000000E+00       1.85221  0.000000E+00  0.000000E+00      -3.70442       58.0434     -0.634584        1       Scatag_D
      22       1694.00       6710.54  0.000000E+00  0.000000E+00       2238.21  0.000000E+00       3065.60       10922.9  0.000000E+00       10552.8       64.7417       22969.5 -1.000000E+10 -1.000000E+10 -1.000000E+10 -1.000000E+10 -1.000000E+10  0.000000E+00  0.000000E+00  0.000000E+00  0.000000E+00  0.000000E+00  0.000000E+00  0.000000E+00  0.000000E+00  0.000000E+00  0.000000E+00  0.000000E+00  0.000000E+00       Funtag_D
      23  1.865073E+05  1.301116E+05       56395.7       84.5487      0.135597      0.825314      0.240756      0.232678    -0.0452445    -0.0399690    0.00761588       1.07684       Opttag_D
      23       59.6469       4.72395       1.00000  0.000000E+00       1.00000  0.000000E+00      0.903877       59.8013       1.00000       10.6242  0.000000E+00       1.86182  0.000000E+00       1.86182  0.000000E+00  0.000000E+00      -3.72363       58.0434     -0.634584        1       Scatag_D
      23       1621.51       6310.79  0.000000E+00  0.000000E+00       2237.11  0.000000E+00       3026.16       10258.1  0.000000E+00       10456.6       59.8660       22425.5 -1.000000E+10 -1.000000E+10 -1.000000E+10 -1.000000E+10 -1.000000E+10  0.000000E+00  0.000000E+00  0.000000E+00  0.000000E+00  0.000000E+00  0.000000E+00  0.000000E+00  0.000000E+00  0.000000E+00  0.000000E+00  0.000000E+00  0.000000E+00       Funtag_D
      24  1.843659E+05  1.302529E+05       54113.0       64.3588      0.138809      0.864814      0.241244      0.233122    -0.0464620    -0.0410819    0.00746389       1.05655       Opttag_D
      24       59.6134       4.74312       1.00000  0.000000E+00       1.00000  0.000000E+00      0.903489       59.7298       1.00000       10.6497  0.000000E+00       1.88333  0.000000E+00       1.88333  0.000000E+00  0.000000E+00      -3.76665       58.0434     -0.634584        1       Scatag_D
      24       1557.43       6075.19  0.000000E+00  0.000000E+00       2234.98  0.000000E+00       2942.55       9542.27  0.000000E+00       10253.4       51.3811       21455.8 -1.000000E+10 -1.000000E+10 -1.000000E+10 -1.000000E+10 -1.000000E+10  0.000000E+00  0.000000E+00  0.000000E+00  0.000000E+00  0.000000E+00  0.000000E+00  0.000000E+00  0.000000E+00  0.000000E+00  0.000000E+00  0.000000E+00  0.000000E+00       Funtag_D
      25  1.831443E+05  1.303783E+05       52766.0       87.8387      0.141938      0.901594      0.241705      0.233530    -0.0475427    -0.0420236    0.00739934       1.06305       Opttag_D
      25       59.5815       4.75950       1.00000  0.000000E+00       1.00000  0.000000E+00      0.903104       59.6505       1.00000       10.6835  0.000000E+00       1.90065  0.000000E+00       1.90065  0.000000E+00  0.000000E+00      -3.80130       58.0434     -0.634584        1       Scatag_D
      25       1530.61       6150.21  0.000000E+00  0.000000E+00       2233.67  0.000000E+00       2881.92       8959.44  0.000000E+00       10073.3       49.3691       20887.5 -1.000000E+10 -1.000000E+10 -1.000000E+10 -1.000000E+10 -1.000000E+10  0.000000E+00  0.000000E+00  0.000000E+00  0.000000E+00  0.000000E+00  0.000000E+00  0.000000E+00  0.000000E+00  0.000000E+00  0.000000E+00  0.000000E+00  0.000000E+00       Funtag_D
      26  1.816141E+05  1.305289E+05       51085.2       71.3219      0.145479      0.944834      0.242246      0.234038    -0.0488412    -0.0431534    0.00725198       1.04808       Opttag_D
      26       59.5440       4.77983       1.00000  0.000000E+00       1.00000  0.000000E+00      0.902675       59.5605       1.00000       10.7188  0.000000E+00       1.92103  0.000000E+00       1.92103  0.000000E+00  0.000000E+00      -3.84206       58.0434     -0.634584        1       Scatag_D
      26       1470.25       5978.20  0.000000E+00  0.000000E+00       2232.32  0.000000E+00       2803.93       8463.12  0.000000E+00       9863.99       48.6756       20224.8 -1.000000E+10 -1.000000E+10 -1.000000E+10 -1.000000E+10 -1.000000E+10  0.000000E+00  0.000000E+00  0.000000E+00  0.000000E+00  0.000000E+00  0.000000E+00  0.000000E+00  0.000000E+00  0.000000E+00  0.000000E+00  0.000000E+00  0.000000E+00       Funtag_D
      27  1.802567E+05  1.307461E+05       49510.6       107.285      0.150623       1.00745      0.243020      0.234774    -0.0507134    -0.0447677    0.00727690       1.05729       Opttag_D
      27       59.4956       4.81047       1.00000  0.000000E+00       1.00000  0.000000E+00      0.902101       59.4394       1.00000       10.7683  0.000000E+00       1.94996  0.000000E+00       1.94996  0.000000E+00  0.000000E+00      -3.89992       58.0434     -0.634584        1       Scatag_D
      27       1480.37       6083.77  0.000000E+00  0.000000E+00       2230.53  0.000000E+00       2698.29       7955.94  0.000000E+00       9574.31       53.1900       19434.2 -1.000000E+10 -1.000000E+10 -1.000000E+10 -1.000000E+10 -1.000000E+10  0.000000E+00  0.000000E+00  0.000000E+00  0.000000E+00  0.000000E+00  0.000000E+00  0.000000E+00  0.000000E+00  0.000000E+00  0.000000E+00  0.000000E+00  0.000000E+00       Funtag_D
      28  1.790052E+05  1.307963E+05       48208.9       59.3079      0.152056       1.02717      0.243222      0.234980    -0.0511457    -0.0451447    0.00708750       1.02883       Opttag_D
      28       59.4815       4.81645       1.00000  0.000000E+00       1.00000  0.000000E+00      0.901886       59.4052       1.00000       10.7894  0.000000E+00       1.95755  0.000000E+00       1.95755  0.000000E+00  0.000000E+00      -3.91511       58.0434     -0.634584        1       Scatag_D
      28       1404.32       5760.69  0.000000E+00  0.000000E+00       2229.23  0.000000E+00       2656.15       7635.52  0.000000E+00       9470.84       54.7698       18997.4 -1.000000E+10 -1.000000E+10 -1.000000E+10 -1.000000E+10 -1.000000E+10  0.000000E+00  0.000000E+00  0.000000E+00  0.000000E+00  0.000000E+00  0.000000E+00  0.000000E+00  0.000000E+00  0.000000E+00  0.000000E+00  0.000000E+00  0.000000E+00       Funtag_D
      29  1.779464E+05  1.308267E+05       47119.7       59.8164      0.153087       1.04387      0.243351      0.235135    -0.0514083    -0.0453849    0.00697198       1.02301       Opttag_D
      29       59.4711       4.81972       1.00000  0.000000E+00       1.00000  0.000000E+00      0.901710       59.3854       1.00000       10.8039  0.000000E+00       1.96308  0.000000E+00       1.96308  0.000000E+00  0.000000E+00      -3.92617       58.0434     -0.634584        1       Scatag_D
      29       1358.91       5695.61  0.000000E+00  0.000000E+00       2227.21  0.000000E+00       2614.00       7363.16  0.000000E+00       9367.95       55.3306       18437.5 -1.000000E+10 -1.000000E+10 -1.000000E+10 -1.000000E+10 -1.000000E+10  0.000000E+00  0.000000E+00  0.000000E+00  0.000000E+00  0.000000E+00  0.000000E+00  0.000000E+00  0.000000E+00  0.000000E+00  0.000000E+00  0.000000E+00  0.000000E+00       Funtag_D
      30  1.767941E+05  1.308949E+05       45899.2       79.4825      0.155133       1.07300      0.243624      0.235484    -0.0519961    -0.0460341    0.00692467       1.02226       Opttag_D
      30       59.4559       4.82921       1.00000  0.000000E+00       1.00000  0.000000E+00      0.901434       59.3535       1.00000       10.8292  0.000000E+00       1.97389  0.000000E+00       1.97389  0.000000E+00  0.000000E+00      -3.94778       58.0434     -0.634584        1       Scatag_D
      30       1340.53       5687.29  0.000000E+00  0.000000E+00       2224.05  0.000000E+00       2558.71       7077.51  0.000000E+00       9209.22       53.0483       17748.8 -1.000000E+10 -1.000000E+10 -1.000000E+10 -1.000000E+10 -1.000000E+10  0.000000E+00  0.000000E+00  0.000000E+00  0.000000E+00  0.000000E+00  0.000000E+00  0.000000E+00  0.000000E+00  0.000000E+00  0.000000E+00  0.000000E+00  0.000000E+00       Funtag_D
      31  1.759830E+05  1.310790E+05       44903.9       113.672      0.159844       1.12829      0.244284      0.236275    -0.0535829    -0.0477776    0.00707723       1.03251       Opttag_D
      31       59.4308       4.85958       1.00000  0.000000E+00       1.00000  0.000000E+00      0.901034       59.2912       1.00000       10.8717  0.000000E+00       1.99834  0.000000E+00       1.99834  0.000000E+00  0.000000E+00      -3.99668       58.0434     -0.634584        1       Scatag_D
      31       1400.25       5801.90  0.000000E+00  0.000000E+00       2222.40  0.000000E+00       2492.31       6691.52  0.000000E+00       8966.54       48.4244       17280.6 -1.000000E+10 -1.000000E+10 -1.000000E+10 -1.000000E+10 -1.000000E+10  0.000000E+00  0.000000E+00  0.000000E+00  0.000000E+00  0.000000E+00  0.000000E+00  0.000000E+00  0.000000E+00  0.000000E+00  0.000000E+00  0.000000E+00  0.000000E+00       Funtag_D
      32  1.749470E+05  1.310959E+05       43851.1       49.9670      0.160194       1.13181      0.244337      0.236378    -0.0537285    -0.0479944    0.00683531      0.995939       Opttag_D
      32       59.4286       4.86268       1.00000  0.000000E+00       1.00000  0.000000E+00      0.901025       59.2888       1.00000       10.8706  0.000000E+00       2.00091  0.000000E+00       2.00091  0.000000E+00  0.000000E+00      -4.00182       58.0434     -0.634584        1       Scatag_D
      32       1306.16       5398.20  0.000000E+00  0.000000E+00       2221.34  0.000000E+00       2472.57       6425.91  0.000000E+00       8940.46       44.3265       17042.1 -1.000000E+10 -1.000000E+10 -1.000000E+10 -1.000000E+10 -1.000000E+10  0.000000E+00  0.000000E+00  0.000000E+00  0.000000E+00  0.000000E+00  0.000000E+00  0.000000E+00  0.000000E+00  0.000000E+00  0.000000E+00  0.000000E+00  0.000000E+00       Funtag_D
      33  1.742335E+05  1.311461E+05       43087.5       46.4517      0.161410       1.14356      0.244512      0.236624    -0.0541607    -0.0485263    0.00671124      0.991379       Opttag_D
      33       59.4216       4.87112       1.00000  0.000000E+00       1.00000  0.000000E+00      0.900955       59.2711       1.00000       10.8778  0.000000E+00       2.00759  0.000000E+00       2.00759  0.000000E+00  0.000000E+00      -4.01517       58.0434     -0.634584        1       Scatag_D
      33       1259.17       5348.88  0.000000E+00  0.000000E+00       2220.18  0.000000E+00       2437.27       6206.67  0.000000E+00       8881.15       40.6387       16693.5 -1.000000E+10 -1.000000E+10 -1.000000E+10 -1.000000E+10 -1.000000E+10  0.000000E+00  0.000000E+00  0.000000E+00  0.000000E+00  0.000000E+00  0.000000E+00  0.000000E+00  0.000000E+00  0.000000E+00  0.000000E+00  0.000000E+00  0.000000E+00       Funtag_D
      34  1.734932E+05  1.312469E+05       42246.3       54.7999      0.164078       1.16881      0.244867      0.237095    -0.0550301    -0.0495198    0.00664648      0.987668       Opttag_D
      34       59.4088       4.88820       1.00000  0.000000E+00       1.00000  0.000000E+00      0.900805       59.2351       1.00000       10.8969  0.000000E+00       2.02039  0.000000E+00       2.02039  0.000000E+00  0.000000E+00      -4.04077       58.0434     -0.634584        1       Scatag_D
      34       1234.99       5308.91  0.000000E+00  0.000000E+00       2218.85  0.000000E+00       2386.10       6052.87  0.000000E+00       8767.25       39.2881       16238.1 -1.000000E+10 -1.000000E+10 -1.000000E+10 -1.000000E+10 -1.000000E+10  0.000000E+00  0.000000E+00  0.000000E+00  0.000000E+00  0.000000E+00  0.000000E+00  0.000000E+00  0.000000E+00  0.000000E+00  0.000000E+00  0.000000E+00  0.000000E+00       Funtag_D
      35  1.727514E+05  1.314968E+05       41254.7       122.407      0.170826       1.22536      0.245727      0.238245    -0.0571838    -0.0518960    0.00682038       1.01634       Opttag_D
      35       59.3869       4.93351       1.00000  0.000000E+00       1.00000  0.000000E+00      0.900595       59.1583       1.00000       10.9327  0.000000E+00       2.05003  0.000000E+00       2.05003  0.000000E+00  0.000000E+00      -4.10006       58.0434     -0.634584        1       Scatag_D
      35       1300.46       5621.58  0.000000E+00  0.000000E+00       2216.08  0.000000E+00       2295.09       5831.96  0.000000E+00       8525.07       41.7242       15422.7 -1.000000E+10 -1.000000E+10 -1.000000E+10 -1.000000E+10 -1.000000E+10  0.000000E+00  0.000000E+00  0.000000E+00  0.000000E+00  0.000000E+00  0.000000E+00  0.000000E+00  0.000000E+00  0.000000E+00  0.000000E+00  0.000000E+00  0.000000E+00       Funtag_D
      36  1.716369E+05  1.315601E+05       40076.7       49.5508      0.172834       1.24024      0.245954      0.238562    -0.0577297    -0.0524942    0.00656840      0.969822       Opttag_D
      36       59.3823       4.94495       1.00000  0.000000E+00       1.00000  0.000000E+00      0.900528       59.1333       1.00000       10.9488  0.000000E+00       2.05705  0.000000E+00       2.05705  0.000000E+00  0.000000E+00      -4.11410       58.0434     -0.634584        1       Scatag_D
      36       1206.14       5118.79  0.000000E+00  0.000000E+00       2215.46  0.000000E+00       2272.69       5564.74  0.000000E+00       8455.90       42.7506       15200.3 -1.000000E+10 -1.000000E+10 -1.000000E+10 -1.000000E+10 -1.000000E+10  0.000000E+00  0.000000E+00  0.000000E+00  0.000000E+00  0.000000E+00  0.000000E+00  0.000000E+00  0.000000E+00  0.000000E+00  0.000000E+00  0.000000E+00  0.000000E+00       Funtag_D
      37  1.709948E+05  1.315915E+05       39403.3       41.8296      0.174158       1.24807      0.246074      0.238728    -0.0580004    -0.0527786    0.00646591      0.964593       Opttag_D
      37       59.3801       4.95067       1.00000  0.000000E+00       1.00000  0.000000E+00      0.900501       59.1163       1.00000       10.9575  0.000000E+00       2.06050  0.000000E+00       2.06050  0.000000E+00  0.000000E+00      -4.12100       58.0434     -0.634584        1       Scatag_D
      37       1168.80       5063.75  0.000000E+00  0.000000E+00       2213.92  0.000000E+00       2252.72       5328.76  0.000000E+00       8397.82       44.2526       14933.3 -1.000000E+10 -1.000000E+10 -1.000000E+10 -1.000000E+10 -1.000000E+10  0.000000E+00  0.000000E+00  0.000000E+00  0.000000E+00  0.000000E+00  0.000000E+00  0.000000E+00  0.000000E+00  0.000000E+00  0.000000E+00  0.000000E+00  0.000000E+00       Funtag_D
      38  1.703026E+05  1.316818E+05       38620.8       49.3437      0.177103       1.26559      0.246387      0.239190    -0.0587782    -0.0536710    0.00641474      0.960467       Opttag_D
      38       59.3784       4.96872       1.00000  0.000000E+00       1.00000  0.000000E+00      0.900500       59.0855       1.00000       10.9709  0.000000E+00       2.07027  0.000000E+00       2.07027  0.000000E+00  0.000000E+00      -4.14054       58.0434     -0.634584        1       Scatag_D
      38       1150.37       5020.52  0.000000E+00  0.000000E+00       2211.38  0.000000E+00       2218.56       5165.08  0.000000E+00       8290.04       45.1425       14519.7 -1.000000E+10 -1.000000E+10 -1.000000E+10 -1.000000E+10 -1.000000E+10  0.000000E+00  0.000000E+00  0.000000E+00  0.000000E+00  0.000000E+00  0.000000E+00  0.000000E+00  0.000000E+00  0.000000E+00  0.000000E+00  0.000000E+00  0.000000E+00       Funtag_D
      39  1.699173E+05  1.318730E+05       38044.4       125.286      0.182961       1.29623      0.247025      0.240211    -0.0604262    -0.0556743    0.00665074       1.00146       Opttag_D
      39       59.3817       5.00897       1.00000  0.000000E+00       1.00000  0.000000E+00      0.900591       59.0287       1.00000       10.9951  0.000000E+00       2.09129  0.000000E+00       2.09129  0.000000E+00  0.000000E+00      -4.18258       58.0434     -0.634584        1       Scatag_D
      39       1236.57       5458.25  0.000000E+00  0.000000E+00       2206.18  0.000000E+00       2157.04       4957.93  0.000000E+00       8092.60       41.3868       13894.4 -1.000000E+10 -1.000000E+10 -1.000000E+10 -1.000000E+10 -1.000000E+10  0.000000E+00  0.000000E+00  0.000000E+00  0.000000E+00  0.000000E+00  0.000000E+00  0.000000E+00  0.000000E+00  0.000000E+00  0.000000E+00  0.000000E+00  0.000000E+00       Funtag_D
      40  1.689559E+05  1.319137E+05       37042.2       44.3934      0.184033       1.30117      0.247153      0.240425    -0.0607775    -0.0561396    0.00633440      0.946912       Opttag_D
      40       59.3837       5.01813       1.00000  0.000000E+00       1.00000  0.000000E+00      0.900641       59.0215       1.00000       10.9950  0.000000E+00       2.09578  0.000000E+00       2.09578  0.000000E+00  0.000000E+00      -4.19156       58.0434     -0.634584        1       Scatag_D
      40       1121.73       4879.81  0.000000E+00  0.000000E+00       2205.42  0.000000E+00       2145.55       4825.05  0.000000E+00       8056.80       39.9500       13767.8 -1.000000E+10 -1.000000E+10 -1.000000E+10 -1.000000E+10 -1.000000E+10  0.000000E+00  0.000000E+00  0.000000E+00  0.000000E+00  0.000000E+00  0.000000E+00  0.000000E+00  0.000000E+00  0.000000E+00  0.000000E+00  0.000000E+00  0.000000E+00       Funtag_D
      41  1.685341E+05  1.319416E+05       36592.5       31.2099      0.184740       1.30337      0.247238      0.240600    -0.0610182    -0.0565154    0.00625517      0.942358       Opttag_D
      41       59.3856       5.02461       1.00000  0.000000E+00       1.00000  0.000000E+00      0.900689       59.0160       1.00000       10.9936  0.000000E+00       2.09943  0.000000E+00       2.09943  0.000000E+00  0.000000E+00      -4.19886       58.0434     -0.634584        1       Scatag_D
      41       1093.85       4832.98  0.000000E+00  0.000000E+00       2204.33  0.000000E+00       2129.39       4667.28  0.000000E+00       8023.82       37.2275       13603.6 -1.000000E+10 -1.000000E+10 -1.000000E+10 -1.000000E+10 -1.000000E+10  0.000000E+00  0.000000E+00  0.000000E+00  0.000000E+00  0.000000E+00  0.000000E+00  0.000000E+00  0.000000E+00  0.000000E+00  0.000000E+00  0.000000E+00  0.000000E+00       Funtag_D
      42  1.680635E+05  1.320143E+05       36049.2       39.3462      0.186807       1.31099      0.247471      0.241023    -0.0616444    -0.0574036    0.00619714      0.941360       Opttag_D
      42       59.3902       5.04104       1.00000  0.000000E+00       1.00000  0.000000E+00      0.900787       58.9985       1.00000       10.9965  0.000000E+00       2.10823  0.000000E+00       2.10823  0.000000E+00  0.000000E+00      -4.21646       58.0434     -0.634584        1       Scatag_D
      42       1073.64       4822.75  0.000000E+00  0.000000E+00       2202.19  0.000000E+00       2098.79       4524.15  0.000000E+00       7946.39       35.2776       13346.0 -1.000000E+10 -1.000000E+10 -1.000000E+10 -1.000000E+10 -1.000000E+10  0.000000E+00  0.000000E+00  0.000000E+00  0.000000E+00  0.000000E+00  0.000000E+00  0.000000E+00  0.000000E+00  0.000000E+00  0.000000E+00  0.000000E+00  0.000000E+00       Funtag_D
      43  1.673853E+05  1.321097E+05       35275.6       55.0790      0.189789       1.32009      0.247771      0.241583    -0.0624667    -0.0586168    0.00610239      0.941877       Opttag_D
      43       59.3989       5.06333       1.00000  0.000000E+00       1.00000  0.000000E+00      0.900955       58.9741       1.00000       10.9990  0.000000E+00       2.11998  0.000000E+00       2.11998  0.000000E+00  0.000000E+00      -4.23996       58.0434     -0.634584        1       Scatag_D
      43       1041.07       4828.05  0.000000E+00  0.000000E+00       2198.77  0.000000E+00       2051.67       4302.61  0.000000E+00       7828.12       37.2528       12988.0 -1.000000E+10 -1.000000E+10 -1.000000E+10 -1.000000E+10 -1.000000E+10  0.000000E+00  0.000000E+00  0.000000E+00  0.000000E+00  0.000000E+00  0.000000E+00  0.000000E+00  0.000000E+00  0.000000E+00  0.000000E+00  0.000000E+00  0.000000E+00       Funtag_D
      44  1.670809E+05  1.323058E+05       34775.1       85.7678      0.195973       1.33922      0.248425      0.242655    -0.0641569    -0.0609914    0.00622523      0.954208       Opttag_D
      44       59.4206       5.10951       1.00000  0.000000E+00       1.00000  0.000000E+00      0.901272       58.9196       1.00000       11.0187  0.000000E+00       2.14170  0.000000E+00       2.14170  0.000000E+00  0.000000E+00      -4.28340       58.0434     -0.634584        1       Scatag_D
      44       1083.40       4955.30  0.000000E+00  0.000000E+00       2194.26  0.000000E+00       1994.09       4096.47  0.000000E+00       7640.28       40.3947       12770.9 -1.000000E+10 -1.000000E+10 -1.000000E+10 -1.000000E+10 -1.000000E+10  0.000000E+00  0.000000E+00  0.000000E+00  0.000000E+00  0.000000E+00  0.000000E+00  0.000000E+00  0.000000E+00  0.000000E+00  0.000000E+00  0.000000E+00  0.000000E+00       Funtag_D
      45  1.664811E+05  1.322752E+05       34205.9       31.0503      0.195263       1.33611      0.248324      0.242509    -0.0638932    -0.0606709    0.00597649      0.927583       Opttag_D
      45       59.4191       5.10272       1.00000  0.000000E+00       1.00000  0.000000E+00      0.901241       58.9234       1.00000       11.0159  0.000000E+00       2.13830  0.000000E+00       2.13830  0.000000E+00  0.000000E+00      -4.27660       58.0434     -0.634584        1       Scatag_D
      45       998.553       4682.62  0.000000E+00  0.000000E+00       2193.69  0.000000E+00       1992.53       4006.64  0.000000E+00       7646.54       40.1438       12645.2 -1.000000E+10 -1.000000E+10 -1.000000E+10 -1.000000E+10 -1.000000E+10  0.000000E+00  0.000000E+00  0.000000E+00  0.000000E+00  0.000000E+00  0.000000E+00  0.000000E+00  0.000000E+00  0.000000E+00  0.000000E+00  0.000000E+00  0.000000E+00       Funtag_D
      46  1.661825E+05  1.322707E+05       33911.8       28.7122      0.195430       1.33564      0.248314      0.242505    -0.0638545    -0.0606651    0.00591864      0.925577       Opttag_D
      46       59.4215       5.10222       1.00000  0.000000E+00       1.00000  0.000000E+00      0.901249       58.9178       1.00000       11.0178  0.000000E+00       2.13746  0.000000E+00       2.13746  0.000000E+00  0.000000E+00      -4.27491       58.0434     -0.634584        1       Scatag_D
      46       979.314       4662.39  0.000000E+00  0.000000E+00       2192.58  0.000000E+00       1985.39       3923.93  0.000000E+00       7629.08       39.4473       12499.6 -1.000000E+10 -1.000000E+10 -1.000000E+10 -1.000000E+10 -1.000000E+10  0.000000E+00  0.000000E+00  0.000000E+00  0.000000E+00  0.000000E+00  0.000000E+00  0.000000E+00  0.000000E+00  0.000000E+00  0.000000E+00  0.000000E+00  0.000000E+00       Funtag_D
      47  1.657398E+05  1.323148E+05       33425.0       38.8038      0.197286       1.33935      0.248470      0.242767    -0.0642342    -0.0612556    0.00588523      0.923585       Opttag_D
      47       59.4328       5.11388       1.00000  0.000000E+00       1.00000  0.000000E+00      0.901352       58.8942       1.00000       11.0269  0.000000E+00       2.14123  0.000000E+00       2.14123  0.000000E+00  0.000000E+00      -4.28245       58.0434     -0.634584        1       Scatag_D
      47       968.291       4642.35  0.000000E+00  0.000000E+00       2189.58  0.000000E+00       1964.90       3821.83  0.000000E+00       7561.29       37.7391       12239.0 -1.000000E+10 -1.000000E+10 -1.000000E+10 -1.000000E+10 -1.000000E+10  0.000000E+00  0.000000E+00  0.000000E+00  0.000000E+00  0.000000E+00  0.000000E+00  0.000000E+00  0.000000E+00  0.000000E+00  0.000000E+00  0.000000E+00  0.000000E+00       Funtag_D
      48  1.652641E+05  1.324412E+05       32822.9       76.0985      0.201825       1.34876      0.248907      0.243471    -0.0653240    -0.0628152    0.00598174      0.932540       Opttag_D
      48       59.4612       5.14659       1.00000  0.000000E+00       1.00000  0.000000E+00      0.901631       58.8424       1.00000       11.0488  0.000000E+00       2.15285  0.000000E+00       2.15285  0.000000E+00  0.000000E+00      -4.30570       58.0434     -0.634584        1       Scatag_D
      48       1000.31       4732.80  0.000000E+00  0.000000E+00       2184.42  0.000000E+00       1924.48       3634.59  0.000000E+00       7428.09       34.7546       11883.5 -1.000000E+10 -1.000000E+10 -1.000000E+10 -1.000000E+10 -1.000000E+10  0.000000E+00  0.000000E+00  0.000000E+00  0.000000E+00  0.000000E+00  0.000000E+00  0.000000E+00  0.000000E+00  0.000000E+00  0.000000E+00  0.000000E+00  0.000000E+00       Funtag_D
      49  1.647104E+05  1.325229E+05       32187.5       39.2314      0.204651       1.35405      0.249182      0.243928    -0.0660286    -0.0637686    0.00584723      0.906742       Opttag_D
      49       59.4811       5.16804       1.00000  0.000000E+00       1.00000  0.000000E+00      0.901821       58.8102       1.00000       11.0634  0.000000E+00       2.16004  0.000000E+00       2.16004  0.000000E+00  0.000000E+00      -4.32007       58.0434     -0.634584        1       Scatag_D
      49       955.827       4474.57  0.000000E+00  0.000000E+00       2180.89  0.000000E+00       1903.88       3537.69  0.000000E+00       7354.41       33.4663       11746.8 -1.000000E+10 -1.000000E+10 -1.000000E+10 -1.000000E+10 -1.000000E+10  0.000000E+00  0.000000E+00  0.000000E+00  0.000000E+00  0.000000E+00  0.000000E+00  0.000000E+00  0.000000E+00  0.000000E+00  0.000000E+00  0.000000E+00  0.000000E+00       Funtag_D
      50  1.643216E+05  1.325675E+05       31754.1       27.7780      0.206043       1.35646      0.249331      0.244172    -0.0664125    -0.0642524    0.00577329      0.899501       Opttag_D
      50       59.4923       5.17986       1.00000  0.000000E+00       1.00000  0.000000E+00      0.901929       58.7944       1.00000       11.0695  0.000000E+00       2.16436  0.000000E+00       2.16436  0.000000E+00  0.000000E+00      -4.32873       58.0434     -0.634584        1       Scatag_D
      50       931.807       4403.39  0.000000E+00  0.000000E+00       2178.82  0.000000E+00       1883.70       3400.73  0.000000E+00       7313.28       33.5290       11608.8 -1.000000E+10 -1.000000E+10 -1.000000E+10 -1.000000E+10 -1.000000E+10  0.000000E+00  0.000000E+00  0.000000E+00  0.000000E+00  0.000000E+00  0.000000E+00  0.000000E+00  0.000000E+00  0.000000E+00  0.000000E+00  0.000000E+00  0.000000E+00       Funtag_D
      51  1.639279E+05  1.326404E+05       31287.5       34.8521      0.208333       1.35996      0.249570      0.244560    -0.0670413    -0.0650407    0.00573533      0.893779       Opttag_D
      51       59.5128       5.19978       1.00000  0.000000E+00       1.00000  0.000000E+00      0.902125       58.7699       1.00000       11.0783  0.000000E+00       2.17138  0.000000E+00       2.17138  0.000000E+00  0.000000E+00      -4.34275       58.0434     -0.634584        1       Scatag_D
      51       919.592       4347.54  0.000000E+00  0.000000E+00       2175.25  0.000000E+00       1851.44       3302.56  0.000000E+00       7244.97       35.0542       11411.1 -1.000000E+10 -1.000000E+10 -1.000000E+10 -1.000000E+10 -1.000000E+10  0.000000E+00  0.000000E+00  0.000000E+00  0.000000E+00  0.000000E+00  0.000000E+00  0.000000E+00  0.000000E+00  0.000000E+00  0.000000E+00  0.000000E+00  0.000000E+00       Funtag_D
      52  1.637331E+05  1.327270E+05       31006.2       61.8715      0.211106       1.36364      0.249858      0.245030    -0.0677871    -0.0659944    0.00578341      0.895680       Opttag_D
      52       59.5415       5.22486       1.00000  0.000000E+00       1.00000  0.000000E+00      0.902396       58.7444       1.00000       11.0858  0.000000E+00       2.17907  0.000000E+00       2.17907  0.000000E+00  0.000000E+00      -4.35813       58.0434     -0.634584        1       Scatag_D
      52       935.074       4366.06  0.000000E+00  0.000000E+00       2170.29  0.000000E+00       1819.74       3280.35  0.000000E+00       7160.76       36.9737       11236.9 -1.000000E+10 -1.000000E+10 -1.000000E+10 -1.000000E+10 -1.000000E+10  0.000000E+00  0.000000E+00  0.000000E+00  0.000000E+00  0.000000E+00  0.000000E+00  0.000000E+00  0.000000E+00  0.000000E+00  0.000000E+00  0.000000E+00  0.000000E+00       Funtag_D
      53  1.633928E+05  1.327131E+05       30679.6       28.2108      0.210802       1.36261      0.249814      0.244981    -0.0676677    -0.0658541    0.00567843      0.884399       Opttag_D
      53       59.5419       5.22191       1.00000  0.000000E+00       1.00000  0.000000E+00      0.902380       58.7447       1.00000       11.0846  0.000000E+00       2.17775  0.000000E+00       2.17775  0.000000E+00  0.000000E+00      -4.35551       58.0434     -0.634584        1       Scatag_D
      53       901.437       4256.77  0.000000E+00  0.000000E+00       2169.44  0.000000E+00       1818.07       3182.64  0.000000E+00       7156.00       37.2229       11158.1 -1.000000E+10 -1.000000E+10 -1.000000E+10 -1.000000E+10 -1.000000E+10  0.000000E+00  0.000000E+00  0.000000E+00  0.000000E+00  0.000000E+00  0.000000E+00  0.000000E+00  0.000000E+00  0.000000E+00  0.000000E+00  0.000000E+00  0.000000E+00       Funtag_D
      54  1.631249E+05  1.327132E+05       30411.7       21.9950      0.211103       1.36214      0.249818      0.245023    -0.0676687    -0.0659030    0.00562343      0.880930       Opttag_D
      54       59.5504       5.22374       1.00000  0.000000E+00       1.00000  0.000000E+00      0.902420       58.7376       1.00000       11.0867  0.000000E+00       2.17707  0.000000E+00       2.17707  0.000000E+00  0.000000E+00      -4.35413       58.0434     -0.634584        1       Scatag_D
      54       884.058       4223.44  0.000000E+00  0.000000E+00       2167.15  0.000000E+00       1813.93       3111.40  0.000000E+00       7130.57       36.9792       11044.1 -1.000000E+10 -1.000000E+10 -1.000000E+10 -1.000000E+10 -1.000000E+10  0.000000E+00  0.000000E+00  0.000000E+00  0.000000E+00  0.000000E+00  0.000000E+00  0.000000E+00  0.000000E+00  0.000000E+00  0.000000E+00  0.000000E+00  0.000000E+00       Funtag_D
      55  1.628954E+05  1.327320E+05       30163.3       26.6189      0.212108       1.36259      0.249891      0.245182    -0.0678310    -0.0661712    0.00560961      0.878211       Opttag_D
      55       59.5668       5.23120       1.00000  0.000000E+00       1.00000  0.000000E+00      0.902516       58.7210       1.00000       11.0939  0.000000E+00       2.17777  0.000000E+00       2.17777  0.000000E+00  0.000000E+00      -4.35554       58.0434     -0.634584        1       Scatag_D
      55       879.720       4197.40  0.000000E+00  0.000000E+00       2163.75  0.000000E+00       1807.34       3063.60  0.000000E+00       7088.24       36.0304       10927.3 -1.000000E+10 -1.000000E+10 -1.000000E+10 -1.000000E+10 -1.000000E+10  0.000000E+00  0.000000E+00  0.000000E+00  0.000000E+00  0.000000E+00  0.000000E+00  0.000000E+00  0.000000E+00  0.000000E+00  0.000000E+00  0.000000E+00  0.000000E+00       Funtag_D
      56  1.627789E+05  1.327864E+05       29992.6       77.4306      0.214895       1.36401      0.250100      0.245617    -0.0682994    -0.0669948    0.00564748      0.884996       Opttag_D
      56       59.6127       5.25236       1.00000  0.000000E+00       1.00000  0.000000E+00      0.902779       58.6759       1.00000       11.1159  0.000000E+00       2.17907  0.000000E+00       2.17907  0.000000E+00  0.000000E+00      -4.35815       58.0434     -0.634584        1       Scatag_D
      56       891.635       4262.52  0.000000E+00  0.000000E+00       2156.23  0.000000E+00       1794.33       3120.41  0.000000E+00       6989.52       33.4145       10744.5 -1.000000E+10 -1.000000E+10 -1.000000E+10 -1.000000E+10 -1.000000E+10  0.000000E+00  0.000000E+00  0.000000E+00  0.000000E+00  0.000000E+00  0.000000E+00  0.000000E+00  0.000000E+00  0.000000E+00  0.000000E+00  0.000000E+00  0.000000E+00       Funtag_D
      57  1.623630E+05  1.328050E+05       29558.0       25.4008      0.215648       1.36467      0.250169      0.245726    -0.0684600    -0.0671760    0.00557237      0.871053       Opttag_D
      57       59.6227       5.25837       1.00000  0.000000E+00       1.00000  0.000000E+00      0.902844       58.6637       1.00000       11.1218  0.000000E+00       2.18094  0.000000E+00       2.18094  0.000000E+00  0.000000E+00      -4.36187       58.0434     -0.634584        1       Scatag_D
      57       868.077       4129.26  0.000000E+00  0.000000E+00       2154.97  0.000000E+00       1789.99       2904.69  0.000000E+00       6968.27       32.9578       10709.7 -1.000000E+10 -1.000000E+10 -1.000000E+10 -1.000000E+10 -1.000000E+10  0.000000E+00  0.000000E+00  0.000000E+00  0.000000E+00  0.000000E+00  0.000000E+00  0.000000E+00  0.000000E+00  0.000000E+00  0.000000E+00  0.000000E+00  0.000000E+00       Funtag_D
      58  1.621971E+05  1.328217E+05       29375.4       18.4305      0.216318       1.36518      0.250229      0.245827    -0.0686038    -0.0673675    0.00552939      0.867789       Opttag_D
      58       59.6328       5.26401       1.00000  0.000000E+00       1.00000  0.000000E+00      0.902905       58.6540       1.00000       11.1265  0.000000E+00       2.18222  0.000000E+00       2.18222  0.000000E+00  0.000000E+00      -4.36443       58.0434     -0.634584        1       Scatag_D
      58       854.737       4098.38  0.000000E+00  0.000000E+00       2154.06  0.000000E+00       1783.28       2852.37  0.000000E+00       6948.86       32.6747       10651.0 -1.000000E+10 -1.000000E+10 -1.000000E+10 -1.000000E+10 -1.000000E+10  0.000000E+00  0.000000E+00  0.000000E+00  0.000000E+00  0.000000E+00  0.000000E+00  0.000000E+00  0.000000E+00  0.000000E+00  0.000000E+00  0.000000E+00  0.000000E+00       Funtag_D
      59  1.619747E+05  1.328626E+05       29112.2       25.5117      0.218000       1.36609      0.250377      0.246071    -0.0689560    -0.0678509    0.00548560      0.864321       Opttag_D
      59       59.6600       5.27852       1.00000  0.000000E+00       1.00000  0.000000E+00      0.903073       58.6309       1.00000       11.1371  0.000000E+00       2.18495  0.000000E+00       2.18495  0.000000E+00  0.000000E+00      -4.36991       58.0434     -0.634584        1       Scatag_D
      59       841.254       4065.69  0.000000E+00  0.000000E+00       2151.64  0.000000E+00       1769.16       2807.88  0.000000E+00       6900.10       32.5955       10543.9 -1.000000E+10 -1.000000E+10 -1.000000E+10 -1.000000E+10 -1.000000E+10  0.000000E+00  0.000000E+00  0.000000E+00  0.000000E+00  0.000000E+00  0.000000E+00  0.000000E+00  0.000000E+00  0.000000E+00  0.000000E+00  0.000000E+00  0.000000E+00       Funtag_D
      60  1.616818E+05  1.329325E+05       28749.3       40.2413      0.220728       1.36686      0.250622      0.246471    -0.0695585    -0.0686835    0.00544572      0.861673       Opttag_D
      60       59.7075       5.30417       1.00000  0.000000E+00       1.00000  0.000000E+00      0.903376       58.6054       1.00000       11.1482  0.000000E+00       2.18934  0.000000E+00       2.18934  0.000000E+00  0.000000E+00      -4.37869       58.0434     -0.634584        1       Scatag_D
      60       829.065       4040.81  0.000000E+00  0.000000E+00       2148.14  0.000000E+00       1744.87       2739.06  0.000000E+00       6820.54       33.6576       10393.2 -1.000000E+10 -1.000000E+10 -1.000000E+10 -1.000000E+10 -1.000000E+10  0.000000E+00  0.000000E+00  0.000000E+00  0.000000E+00  0.000000E+00  0.000000E+00  0.000000E+00  0.000000E+00  0.000000E+00  0.000000E+00  0.000000E+00  0.000000E+00       Funtag_D
      61  1.614194E+05  1.330401E+05       28379.4       35.2166      0.224972       1.37728      0.251008      0.247127    -0.0704862    -0.0699077    0.00548137      0.857293       Opttag_D
      61       59.7801       5.34357       1.00000  0.000000E+00       1.00000  0.000000E+00      0.903841       58.5583       1.00000       11.1709  0.000000E+00       2.19637  0.000000E+00       2.19637  0.000000E+00  0.000000E+00      -4.39274       58.0434     -0.634584        1       Scatag_D
      61       839.956       3999.83  0.000000E+00  0.000000E+00       2143.59  0.000000E+00       1716.85       2636.37  0.000000E+00       6715.10       35.0858       10292.6 -1.000000E+10 -1.000000E+10 -1.000000E+10 -1.000000E+10 -1.000000E+10  0.000000E+00  0.000000E+00  0.000000E+00  0.000000E+00  0.000000E+00  0.000000E+00  0.000000E+00  0.000000E+00  0.000000E+00  0.000000E+00  0.000000E+00  0.000000E+00       Funtag_D
      62  1.612138E+05  1.330509E+05       28162.9       19.2093      0.225522       1.37726      0.251050      0.247223    -0.0705796    -0.0700821    0.00543303      0.854837       Opttag_D
      62       59.7923       5.34879       1.00000  0.000000E+00       1.00000  0.000000E+00      0.903901       58.5567       1.00000       11.1732  0.000000E+00       2.19660  0.000000E+00       2.19660  0.000000E+00  0.000000E+00      -4.39320       58.0434     -0.634584        1       Scatag_D
      62       825.208       3976.95  0.000000E+00  0.000000E+00       2142.27  0.000000E+00       1709.54       2548.80  0.000000E+00       6695.47       34.8448       10229.8 -1.000000E+10 -1.000000E+10 -1.000000E+10 -1.000000E+10 -1.000000E+10  0.000000E+00  0.000000E+00  0.000000E+00  0.000000E+00  0.000000E+00  0.000000E+00  0.000000E+00  0.000000E+00  0.000000E+00  0.000000E+00  0.000000E+00  0.000000E+00       Funtag_D
      63  1.610149E+05  1.330677E+05       27947.2       21.6908      0.226483       1.37641      0.251116      0.247382    -0.0707237    -0.0703796    0.00538464      0.853274       Opttag_D
      63       59.8138       5.35734       1.00000  0.000000E+00       1.00000  0.000000E+00      0.904001       58.5503       1.00000       11.1793  0.000000E+00       2.19669  0.000000E+00       2.19669  0.000000E+00  0.000000E+00      -4.39338       58.0434     -0.634584        1       Scatag_D
      63       810.574       3962.42  0.000000E+00  0.000000E+00       2139.54  0.000000E+00       1698.70       2510.06  0.000000E+00       6663.06       34.2270       10128.6 -1.000000E+10 -1.000000E+10 -1.000000E+10 -1.000000E+10 -1.000000E+10  0.000000E+00  0.000000E+00  0.000000E+00  0.000000E+00  0.000000E+00  0.000000E+00  0.000000E+00  0.000000E+00  0.000000E+00  0.000000E+00  0.000000E+00  0.000000E+00       Funtag_D
      64  1.608480E+05  1.331087E+05       27739.4       25.7847      0.228393       1.37972      0.251270      0.247681    -0.0710771    -0.0709381    0.00537266      0.851374       Opttag_D
      64       59.8518       5.37484       1.00000  0.000000E+00       1.00000  0.000000E+00      0.904199       58.5352       1.00000       11.1922  0.000000E+00       2.19828  0.000000E+00       2.19828  0.000000E+00  0.000000E+00      -4.39656       58.0434     -0.634584        1       Scatag_D
      64       806.970       3944.79  0.000000E+00  0.000000E+00       2135.53  0.000000E+00       1685.94       2484.50  0.000000E+00       6614.64       33.6930       10033.3 -1.000000E+10 -1.000000E+10 -1.000000E+10 -1.000000E+10 -1.000000E+10  0.000000E+00  0.000000E+00  0.000000E+00  0.000000E+00  0.000000E+00  0.000000E+00  0.000000E+00  0.000000E+00  0.000000E+00  0.000000E+00  0.000000E+00  0.000000E+00       Funtag_D
      65  1.606215E+05  1.331849E+05       27436.6       23.6808      0.231798       1.38681      0.251554      0.248191    -0.0717341    -0.0719586    0.00536140      0.847995       Opttag_D
      65       59.9178       5.40615       1.00000  0.000000E+00       1.00000  0.000000E+00      0.904549       58.5068       1.00000       11.2183  0.000000E+00       2.20146  0.000000E+00       2.20146  0.000000E+00  0.000000E+00      -4.40293       58.0434     -0.634584        1       Scatag_D
      65       803.591       3913.54  0.000000E+00  0.000000E+00       2129.13  0.000000E+00       1668.95       2412.44  0.000000E+00       6541.61       33.0995       9934.28 -1.000000E+10 -1.000000E+10 -1.000000E+10 -1.000000E+10 -1.000000E+10  0.000000E+00  0.000000E+00  0.000000E+00  0.000000E+00  0.000000E+00  0.000000E+00  0.000000E+00  0.000000E+00  0.000000E+00  0.000000E+00  0.000000E+00  0.000000E+00       Funtag_D
      66  1.604288E+05  1.332436E+05       27185.2       22.2279      0.234275       1.39238      0.251770      0.248553    -0.0722402    -0.0727104    0.00531017      0.845359       Opttag_D
      66       59.9664       5.42993       1.00000  0.000000E+00       1.00000  0.000000E+00      0.904811       58.4888       1.00000       11.2372  0.000000E+00       2.20371  0.000000E+00       2.20371  0.000000E+00  0.000000E+00      -4.40743       58.0434     -0.634584        1       Scatag_D
      66       788.308       3889.25  0.000000E+00  0.000000E+00       2124.46  0.000000E+00       1657.80       2334.11  0.000000E+00       6494.39       32.9357       9863.92 -1.000000E+10 -1.000000E+10 -1.000000E+10 -1.000000E+10 -1.000000E+10  0.000000E+00  0.000000E+00  0.000000E+00  0.000000E+00  0.000000E+00  0.000000E+00  0.000000E+00  0.000000E+00  0.000000E+00  0.000000E+00  0.000000E+00  0.000000E+00       Funtag_D
      67  1.602421E+05  1.333087E+05       26933.5       20.2100      0.236886       1.39881      0.252009      0.248918    -0.0728012    -0.0734670    0.00524981      0.842624       Opttag_D
      67       60.0187       5.45595       1.00000  0.000000E+00       1.00000  0.000000E+00      0.905088       58.4740       1.00000       11.2583  0.000000E+00       2.20664  0.000000E+00       2.20664  0.000000E+00  0.000000E+00      -4.41327       58.0434     -0.634584        1       Scatag_D
      67       770.487       3864.13  0.000000E+00  0.000000E+00       2119.47  0.000000E+00       1646.22       2274.37  0.000000E+00       6448.45       33.1909       9777.15 -1.000000E+10 -1.000000E+10 -1.000000E+10 -1.000000E+10 -1.000000E+10  0.000000E+00  0.000000E+00  0.000000E+00  0.000000E+00  0.000000E+00  0.000000E+00  0.000000E+00  0.000000E+00  0.000000E+00  0.000000E+00  0.000000E+00  0.000000E+00       Funtag_D
      68  1.600922E+05  1.333486E+05       26743.7       21.3741      0.238397       1.40241      0.252155      0.249115    -0.0731449    -0.0738951    0.00519464      0.841277       Opttag_D
      68       60.0524       5.47255       1.00000  0.000000E+00       1.00000  0.000000E+00      0.905259       58.4716       1.00000       11.2692  0.000000E+00       2.20782  0.000000E+00       2.20782  0.000000E+00  0.000000E+00      -4.41565       58.0434     -0.634584        1       Scatag_D
      68       754.379       3851.78  0.000000E+00  0.000000E+00       2116.00  0.000000E+00       1637.76       2236.71  0.000000E+00       6420.52       33.5687       9692.97 -1.000000E+10 -1.000000E+10 -1.000000E+10 -1.000000E+10 -1.000000E+10  0.000000E+00  0.000000E+00  0.000000E+00  0.000000E+00  0.000000E+00  0.000000E+00  0.000000E+00  0.000000E+00  0.000000E+00  0.000000E+00  0.000000E+00  0.000000E+00       Funtag_D
      69  1.599155E+05  1.334045E+05       26511.0       19.1471      0.240560       1.40724      0.252359      0.249401    -0.0736274    -0.0745257    0.00514624      0.839866       Opttag_D
      69       60.1015       5.49628       1.00000  0.000000E+00       1.00000  0.000000E+00      0.905499       58.4663       1.00000       11.2872  0.000000E+00       2.20907  0.000000E+00       2.20907  0.000000E+00  0.000000E+00      -4.41815       58.0434     -0.634584        1       Scatag_D
      69       740.386       3838.87  0.000000E+00  0.000000E+00       2111.45  0.000000E+00       1627.15       2182.30  0.000000E+00       6382.63       33.8574       9594.33 -1.000000E+10 -1.000000E+10 -1.000000E+10 -1.000000E+10 -1.000000E+10  0.000000E+00  0.000000E+00  0.000000E+00  0.000000E+00  0.000000E+00  0.000000E+00  0.000000E+00  0.000000E+00  0.000000E+00  0.000000E+00  0.000000E+00  0.000000E+00       Funtag_D
      70  1.597585E+05  1.334720E+05       26286.5       23.9946      0.243216       1.41283      0.252613      0.249770    -0.0742086    -0.0752902    0.00513703      0.838046       Opttag_D
      70       60.1621       5.52524       1.00000  0.000000E+00       1.00000  0.000000E+00      0.905777       58.4573       1.00000       11.3129  0.000000E+00       2.21072  0.000000E+00       2.21072  0.000000E+00  0.000000E+00      -4.42144       58.0434     -0.634584        1       Scatag_D
      70       737.738       3822.25  0.000000E+00  0.000000E+00       2106.64  0.000000E+00       1615.05       2133.46  0.000000E+00       6337.90       33.6288       9499.85 -1.000000E+10 -1.000000E+10 -1.000000E+10 -1.000000E+10 -1.000000E+10  0.000000E+00  0.000000E+00  0.000000E+00  0.000000E+00  0.000000E+00  0.000000E+00  0.000000E+00  0.000000E+00  0.000000E+00  0.000000E+00  0.000000E+00  0.000000E+00       Funtag_D
      71  1.596144E+05  1.335210E+05       26093.4       19.1477      0.245184       1.41710      0.252798      0.250054    -0.0746314    -0.0758911    0.00510924      0.835690       Opttag_D
      71       60.2069       5.54631       1.00000  0.000000E+00       1.00000  0.000000E+00      0.905973       58.4454       1.00000       11.3351  0.000000E+00       2.21183  0.000000E+00       2.21183  0.000000E+00  0.000000E+00      -4.42365       58.0434     -0.634584        1       Scatag_D
      71       729.780       3800.79  0.000000E+00  0.000000E+00       2103.69  0.000000E+00       1605.24       2081.54  0.000000E+00       6306.46       33.1865       9432.69 -1.000000E+10 -1.000000E+10 -1.000000E+10 -1.000000E+10 -1.000000E+10  0.000000E+00  0.000000E+00  0.000000E+00  0.000000E+00  0.000000E+00  0.000000E+00  0.000000E+00  0.000000E+00  0.000000E+00  0.000000E+00  0.000000E+00  0.000000E+00       Funtag_D
      72  1.594729E+05  1.335787E+05       25894.2       16.8194      0.247458       1.42251      0.253022      0.250401    -0.0751283    -0.0766272    0.00508189      0.832750       Opttag_D
      72       60.2585       5.57096       1.00000  0.000000E+00       1.00000  0.000000E+00      0.906200       58.4302       1.00000       11.3610  0.000000E+00       2.21326  0.000000E+00       2.21326  0.000000E+00  0.000000E+00      -4.42652       58.0434     -0.634584        1       Scatag_D
      72       721.986       3774.09  0.000000E+00  0.000000E+00       2100.92  0.000000E+00       1593.61       2039.65  0.000000E+00       6272.85       32.6916       9358.39 -1.000000E+10 -1.000000E+10 -1.000000E+10 -1.000000E+10 -1.000000E+10  0.000000E+00  0.000000E+00  0.000000E+00  0.000000E+00  0.000000E+00  0.000000E+00  0.000000E+00  0.000000E+00  0.000000E+00  0.000000E+00  0.000000E+00  0.000000E+00       Funtag_D
      73  1.593420E+05  1.336617E+05       25680.2       24.1758      0.250588       1.43103      0.253334      0.250880    -0.0758441    -0.0776395    0.00505831      0.829691       Opttag_D
      73       60.3308       5.60601       1.00000  0.000000E+00       1.00000  0.000000E+00      0.906525       58.4097       1.00000       11.3974  0.000000E+00       2.21636  0.000000E+00       2.21636  0.000000E+00  0.000000E+00      -4.43272       58.0434     -0.634584        1       Scatag_D
      73       715.301       3746.42  0.000000E+00  0.000000E+00       2097.27  0.000000E+00       1578.70       2012.41  0.000000E+00       6231.06       32.6683       9266.42 -1.000000E+10 -1.000000E+10 -1.000000E+10 -1.000000E+10 -1.000000E+10  0.000000E+00  0.000000E+00  0.000000E+00  0.000000E+00  0.000000E+00  0.000000E+00  0.000000E+00  0.000000E+00  0.000000E+00  0.000000E+00  0.000000E+00  0.000000E+00       Funtag_D
      74  1.592155E+05  1.337416E+05       25473.9       18.1173      0.253384       1.43924      0.253625      0.251301    -0.0765328    -0.0785580    0.00504038      0.827333       Opttag_D
      74       60.3969       5.63954       1.00000  0.000000E+00       1.00000  0.000000E+00      0.906836       58.3989       1.00000       11.4265  0.000000E+00       2.21916  0.000000E+00       2.21916  0.000000E+00  0.000000E+00      -4.43832       58.0434     -0.634584        1       Scatag_D
      74       710.241       3725.16  0.000000E+00  0.000000E+00       2094.20  0.000000E+00       1566.43       1951.04  0.000000E+00       6197.56       32.9399       9196.28 -1.000000E+10 -1.000000E+10 -1.000000E+10 -1.000000E+10 -1.000000E+10  0.000000E+00  0.000000E+00  0.000000E+00  0.000000E+00  0.000000E+00  0.000000E+00  0.000000E+00  0.000000E+00  0.000000E+00  0.000000E+00  0.000000E+00  0.000000E+00       Funtag_D
      75  1.591089E+05  1.337889E+05       25319.9       15.1021      0.254944       1.44487      0.253792      0.251537    -0.0769408    -0.0790785    0.00501662      0.825290       Opttag_D
      75       60.4364       5.65964       1.00000  0.000000E+00       1.00000  0.000000E+00      0.907023       58.3956       1.00000       11.4421  0.000000E+00       2.22087  0.000000E+00       2.22087  0.000000E+00  0.000000E+00      -4.44174       58.0434     -0.634584        1       Scatag_D
      75       703.560       3706.77  0.000000E+00  0.000000E+00       2092.26  0.000000E+00       1557.84       1910.65  0.000000E+00       6177.40       33.1774       9138.28 -1.000000E+10 -1.000000E+10 -1.000000E+10 -1.000000E+10 -1.000000E+10  0.000000E+00  0.000000E+00  0.000000E+00  0.000000E+00  0.000000E+00  0.000000E+00  0.000000E+00  0.000000E+00  0.000000E+00  0.000000E+00  0.000000E+00  0.000000E+00       Funtag_D
      76  1.589786E+05  1.338707E+05       25107.9       19.3447      0.257721       1.45482      0.254092      0.251949    -0.0776453    -0.0799637    0.00499190      0.822570       Opttag_D
      76       60.5074       5.69498       1.00000  0.000000E+00       1.00000  0.000000E+00      0.907339       58.3878       1.00000       11.4747  0.000000E+00       2.22315  0.000000E+00       2.22315  0.000000E+00  0.000000E+00      -4.44631       58.0434     -0.634584        1       Scatag_D
      76       696.642       3682.39  0.000000E+00  0.000000E+00       2088.87  0.000000E+00       1542.95       1881.35  0.000000E+00       6141.89       33.0224       9040.79 -1.000000E+10 -1.000000E+10 -1.000000E+10 -1.000000E+10 -1.000000E+10  0.000000E+00  0.000000E+00  0.000000E+00  0.000000E+00  0.000000E+00  0.000000E+00  0.000000E+00  0.000000E+00  0.000000E+00  0.000000E+00  0.000000E+00  0.000000E+00       Funtag_D
      77  1.588840E+05  1.339792E+05       24904.8       26.5043      0.261409       1.46760      0.254498      0.252504    -0.0785808    -0.0811462    0.00497932      0.820687       Opttag_D
      77       60.6009       5.74186       1.00000  0.000000E+00       1.00000  0.000000E+00      0.907743       58.3747       1.00000       11.5222  0.000000E+00       2.22596  0.000000E+00       2.22596  0.000000E+00  0.000000E+00      -4.45192       58.0434     -0.634584        1       Scatag_D
      77       693.137       3665.55  0.000000E+00  0.000000E+00       2085.30  0.000000E+00       1525.94       1864.34  0.000000E+00       6099.37       32.6401       8938.52 -1.000000E+10 -1.000000E+10 -1.000000E+10 -1.000000E+10 -1.000000E+10  0.000000E+00  0.000000E+00  0.000000E+00  0.000000E+00  0.000000E+00  0.000000E+00  0.000000E+00  0.000000E+00  0.000000E+00  0.000000E+00  0.000000E+00  0.000000E+00       Funtag_D
      78  1.587673E+05  1.339890E+05       24778.3       15.7287      0.261849       1.46962      0.254539      0.252576    -0.0786652    -0.0812601    0.00495291      0.818918       Opttag_D
      78       60.6147       5.74713       1.00000  0.000000E+00       1.00000  0.000000E+00      0.907776       58.3700       1.00000       11.5327  0.000000E+00       2.22537  0.000000E+00       2.22537  0.000000E+00  0.000000E+00      -4.45075       58.0434     -0.634584        1       Scatag_D
      78       685.802       3649.76  0.000000E+00  0.000000E+00       2084.53  0.000000E+00       1522.14       1812.54  0.000000E+00       6089.47       32.1208       8901.96 -1.000000E+10 -1.000000E+10 -1.000000E+10 -1.000000E+10 -1.000000E+10  0.000000E+00  0.000000E+00  0.000000E+00  0.000000E+00  0.000000E+00  0.000000E+00  0.000000E+00  0.000000E+00  0.000000E+00  0.000000E+00  0.000000E+00  0.000000E+00       Funtag_D
      79  1.586436E+05  1.340215E+05       24622.1       14.8327      0.263159       1.47469      0.254672      0.252785    -0.0789454    -0.0816329    0.00492455      0.817087       Opttag_D
      79       60.6516       5.76287       1.00000  0.000000E+00       1.00000  0.000000E+00      0.907885       58.3576       1.00000       11.5595  0.000000E+00       2.22498  0.000000E+00       2.22498  0.000000E+00  0.000000E+00      -4.44997       58.0434     -0.634584        1       Scatag_D
      79       677.973       3633.46  0.000000E+00  0.000000E+00       2083.01  0.000000E+00       1513.86       1779.13  0.000000E+00       6067.45       31.5616       8835.67 -1.000000E+10 -1.000000E+10 -1.000000E+10 -1.000000E+10 -1.000000E+10  0.000000E+00  0.000000E+00  0.000000E+00  0.000000E+00  0.000000E+00  0.000000E+00  0.000000E+00  0.000000E+00  0.000000E+00  0.000000E+00  0.000000E+00  0.000000E+00       Funtag_D
      80  1.585458E+05  1.340875E+05       24458.3       21.5743      0.265503       1.48359      0.254925      0.253147    -0.0795141    -0.0823363    0.00488972      0.814959       Opttag_D
      80       60.7156       5.79289       1.00000  0.000000E+00       1.00000  0.000000E+00      0.908122       58.3433       1.00000       11.5983  0.000000E+00       2.22610  0.000000E+00       2.22610  0.000000E+00  0.000000E+00      -4.45220       58.0434     -0.634584        1       Scatag_D
      80       668.415       3614.56  0.000000E+00  0.000000E+00       2081.13  0.000000E+00       1501.85       1764.02  0.000000E+00       6036.55       31.5617       8760.24 -1.000000E+10 -1.000000E+10 -1.000000E+10 -1.000000E+10 -1.000000E+10  0.000000E+00  0.000000E+00  0.000000E+00  0.000000E+00  0.000000E+00  0.000000E+00  0.000000E+00  0.000000E+00  0.000000E+00  0.000000E+00  0.000000E+00  0.000000E+00       Funtag_D
      81  1.584320E+05  1.341643E+05       24267.7       15.8500      0.268210       1.49415      0.255221      0.253567    -0.0801765    -0.0831485    0.00487043      0.813235       Opttag_D
      81       60.7905       5.82800       1.00000  0.000000E+00       1.00000  0.000000E+00      0.908393       58.3252       1.00000       11.6455  0.000000E+00       2.22773  0.000000E+00       2.22773  0.000000E+00  0.000000E+00      -4.45546       58.0434     -0.634584        1       Scatag_D
      81       663.151       3599.28  0.000000E+00  0.000000E+00       2079.39  0.000000E+00       1488.24       1716.93  0.000000E+00       6002.87       31.8117       8685.99 -1.000000E+10 -1.000000E+10 -1.000000E+10 -1.000000E+10 -1.000000E+10  0.000000E+00  0.000000E+00  0.000000E+00  0.000000E+00  0.000000E+00  0.000000E+00  0.000000E+00  0.000000E+00  0.000000E+00  0.000000E+00  0.000000E+00  0.000000E+00       Funtag_D

 An atom has moved more than     1.50 A from the initial/last
 position - if this was an accepted trial point, probably need to update the
 non-bonded contact list.

      82  1.583358E+05  1.342050E+05       24130.8       14.8599      0.269591       1.50034      0.255378      0.253762    -0.0805271    -0.0835332    0.00484169      0.812069       Opttag_D
      82       60.8328       5.84745       1.00000  0.000000E+00       1.00000  0.000000E+00      0.908545       58.3191       1.00000       11.6694  0.000000E+00       2.22854  0.000000E+00       2.22854  0.000000E+00  0.000000E+00      -4.45708       58.0434     -0.634584        1       Scatag_D
      82       655.350       3588.96  0.000000E+00  0.000000E+00       2078.38  0.000000E+00       1479.57       1681.79  0.000000E+00       5982.81       31.9266       8632.02 -1.000000E+10 -1.000000E+10 -1.000000E+10 -1.000000E+10 -1.000000E+10  0.000000E+00  0.000000E+00  0.000000E+00  0.000000E+00  0.000000E+00  0.000000E+00  0.000000E+00  0.000000E+00  0.000000E+00  0.000000E+00  0.000000E+00  0.000000E+00       Funtag_D

 An atom has moved more than     1.50 A from the initial/last
 position - need to update the non-bonded contact list.


 ############################################################################################################
 ###################################################### setting up contact terms
 ############################################################################################################

      83  1.582132E+05  1.342703E+05       23942.9       18.1296      0.271798       1.53892      0.255628      0.254056    -0.0810899    -0.0841193    0.00481649      0.810539       Opttag_D
      83       60.9053       5.87996       1.00000  0.000000E+00       1.00000  0.000000E+00      0.908800       58.3120       1.00000       11.7082  0.000000E+00       2.22928  0.000000E+00       2.22928  0.000000E+00  0.000000E+00      -4.45855       58.0434     -0.634584        1       Scatag_D
      83       648.545       3575.46  0.000000E+00  0.000000E+00       2076.73  0.000000E+00       1466.26       1647.16  0.000000E+00       5948.21       31.8266       8548.69 -1.000000E+10 -1.000000E+10 -1.000000E+10 -1.000000E+10 -1.000000E+10  0.000000E+00  0.000000E+00  0.000000E+00  0.000000E+00  0.000000E+00  0.000000E+00  0.000000E+00  0.000000E+00  0.000000E+00  0.000000E+00  0.000000E+00  0.000000E+00       Funtag_D
      84  1.581221E+05  1.342972E+05       23825.0       17.4584      0.272797       1.57491      0.255734      0.254163    -0.0813214    -0.0843254    0.00479703      0.809466       Opttag_D
      84       60.9431       5.89537       1.00000  0.000000E+00       1.00000  0.000000E+00      0.908917       58.3111       1.00000       11.7282  0.000000E+00       2.22855  0.000000E+00       2.22855  0.000000E+00  0.000000E+00      -4.45710       58.0434     -0.634584        1       Scatag_D
      84       643.314       3565.99  0.000000E+00  0.000000E+00       2075.45  0.000000E+00       1459.00       1623.77  0.000000E+00       5926.73       31.4071       8499.30 -1.000000E+10 -1.000000E+10 -1.000000E+10 -1.000000E+10 -1.000000E+10  0.000000E+00  0.000000E+00  0.000000E+00  0.000000E+00  0.000000E+00  0.000000E+00  0.000000E+00  0.000000E+00  0.000000E+00  0.000000E+00  0.000000E+00  0.000000E+00       Funtag_D
      85  1.580336E+05  1.343110E+05       23722.6       14.6000      0.273408       1.60392      0.255793      0.254223    -0.0814401    -0.0844356    0.00478494      0.808630       Opttag_D
      85       60.9699       5.90507       1.00000  0.000000E+00       1.00000  0.000000E+00      0.908991       58.3107       1.00000       11.7421  0.000000E+00       2.22710  0.000000E+00       2.22710  0.000000E+00  0.000000E+00      -4.45420       58.0434     -0.634584        1       Scatag_D
      85       640.075       3558.63  0.000000E+00  0.000000E+00       2074.18  0.000000E+00       1453.06       1599.30  0.000000E+00       5909.87       30.8729       8456.64 -1.000000E+10 -1.000000E+10 -1.000000E+10 -1.000000E+10 -1.000000E+10  0.000000E+00  0.000000E+00  0.000000E+00  0.000000E+00  0.000000E+00  0.000000E+00  0.000000E+00  0.000000E+00  0.000000E+00  0.000000E+00  0.000000E+00  0.000000E+00       Funtag_D
      86  1.579270E+05  1.343523E+05       23574.7       20.0559      0.275157       1.67555      0.255962      0.254434    -0.0817967    -0.0848392    0.00479133      0.807268       Opttag_D
      86       61.0366       5.93078       1.00000  0.000000E+00       1.00000  0.000000E+00      0.909191       58.3059       1.00000       11.7789  0.000000E+00       2.22460  0.000000E+00       2.22460  0.000000E+00  0.000000E+00      -4.44920       58.0434     -0.634584        1       Scatag_D
      86       641.786       3546.65  0.000000E+00  0.000000E+00       2071.60  0.000000E+00       1440.37       1589.09  0.000000E+00       5877.58       30.3382       8377.25 -1.000000E+10 -1.000000E+10 -1.000000E+10 -1.000000E+10 -1.000000E+10  0.000000E+00  0.000000E+00  0.000000E+00  0.000000E+00  0.000000E+00  0.000000E+00  0.000000E+00  0.000000E+00  0.000000E+00  0.000000E+00  0.000000E+00  0.000000E+00       Funtag_D
      87  1.578510E+05  1.343902E+05       23460.7       20.9313      0.276754       1.74056      0.256114      0.254641    -0.0821236    -0.0852468    0.00480344      0.806680       Opttag_D
      87       61.0946       5.95359       1.00000  0.000000E+00       1.00000  0.000000E+00      0.909371       58.2988       1.00000       11.8126  0.000000E+00       2.22302  0.000000E+00       2.22302  0.000000E+00  0.000000E+00      -4.44603       58.0434     -0.634584        1       Scatag_D
      87       645.035       3541.49  0.000000E+00  0.000000E+00       2069.21  0.000000E+00       1430.69       1574.40  0.000000E+00       5852.37       30.3529       8317.21 -1.000000E+10 -1.000000E+10 -1.000000E+10 -1.000000E+10 -1.000000E+10  0.000000E+00  0.000000E+00  0.000000E+00  0.000000E+00  0.000000E+00  0.000000E+00  0.000000E+00  0.000000E+00  0.000000E+00  0.000000E+00  0.000000E+00  0.000000E+00       Funtag_D
      88  1.577811E+05  1.343958E+05       23385.3       13.4991      0.277002       1.75111      0.256137      0.254679    -0.0821718    -0.0853383    0.00478103      0.805635       Opttag_D
      88       61.1030       5.95693       1.00000  0.000000E+00       1.00000  0.000000E+00      0.909404       58.2971       1.00000       11.8166  0.000000E+00       2.22307  0.000000E+00       2.22307  0.000000E+00  0.000000E+00      -4.44614       58.0434     -0.634584        1       Scatag_D
      88       639.030       3532.32  0.000000E+00  0.000000E+00       2068.52  0.000000E+00       1428.16       1546.34  0.000000E+00       5848.17       30.2719       8292.47 -1.000000E+10 -1.000000E+10 -1.000000E+10 -1.000000E+10 -1.000000E+10  0.000000E+00  0.000000E+00  0.000000E+00  0.000000E+00  0.000000E+00  0.000000E+00  0.000000E+00  0.000000E+00  0.000000E+00  0.000000E+00  0.000000E+00  0.000000E+00       Funtag_D
      89  1.576919E+05  1.344214E+05       23270.5       11.2363      0.278018       1.79254      0.256235      0.254822    -0.0823923    -0.0856615    0.00475983      0.804392       Opttag_D
      89       61.1397       5.97195       1.00000  0.000000E+00       1.00000  0.000000E+00      0.909543       58.2946       1.00000       11.8337  0.000000E+00       2.22303  0.000000E+00       2.22303  0.000000E+00  0.000000E+00      -4.44606       58.0434     -0.634584        1       Scatag_D
      89       633.377       3521.43  0.000000E+00  0.000000E+00       2066.40  0.000000E+00       1422.21       1527.40  0.000000E+00       5832.87       30.3851       8236.41 -1.000000E+10 -1.000000E+10 -1.000000E+10 -1.000000E+10 -1.000000E+10  0.000000E+00  0.000000E+00  0.000000E+00  0.000000E+00  0.000000E+00  0.000000E+00  0.000000E+00  0.000000E+00  0.000000E+00  0.000000E+00  0.000000E+00  0.000000E+00       Funtag_D
      90  1.576318E+05  1.344522E+05       23179.6       16.8380      0.279244       1.84308      0.256353      0.254979    -0.0826576    -0.0859997    0.00474122      0.803536       Opttag_D
      90       61.1875       5.99121       1.00000  0.000000E+00       1.00000  0.000000E+00      0.909721       58.2953       1.00000       11.8541  0.000000E+00       2.22230  0.000000E+00       2.22230  0.000000E+00  0.000000E+00      -4.44460       58.0434     -0.634584        1       Scatag_D
      90       628.433       3513.94  0.000000E+00  0.000000E+00       2063.68  0.000000E+00       1416.98       1522.40  0.000000E+00       5813.35       30.3352       8190.50 -1.000000E+10 -1.000000E+10 -1.000000E+10 -1.000000E+10 -1.000000E+10  0.000000E+00  0.000000E+00  0.000000E+00  0.000000E+00  0.000000E+00  0.000000E+00  0.000000E+00  0.000000E+00  0.000000E+00  0.000000E+00  0.000000E+00  0.000000E+00       Funtag_D
      91  1.575599E+05  1.344750E+05       23085.0       12.1833      0.280160       1.87902      0.256440      0.255103    -0.0828539    -0.0862826    0.00471156      0.802353       Opttag_D
      91       61.2266       6.00668       1.00000  0.000000E+00       1.00000  0.000000E+00      0.909878       58.2983       1.00000       11.8671  0.000000E+00       2.22144  0.000000E+00       2.22144  0.000000E+00  0.000000E+00      -4.44288       58.0434     -0.634584        1       Scatag_D
      91       620.596       3503.60  0.000000E+00  0.000000E+00       2061.01  0.000000E+00       1413.88       1502.78  0.000000E+00       5797.53       30.1491       8155.41 -1.000000E+10 -1.000000E+10 -1.000000E+10 -1.000000E+10 -1.000000E+10  0.000000E+00  0.000000E+00  0.000000E+00  0.000000E+00  0.000000E+00  0.000000E+00  0.000000E+00  0.000000E+00  0.000000E+00  0.000000E+00  0.000000E+00  0.000000E+00       Funtag_D
      92  1.574971E+05  1.344844E+05       23012.6       12.5792      0.280650       1.89417      0.256475      0.255162    -0.0829353    -0.0864251    0.00468289      0.801219       Opttag_D
      92       61.2528       6.01580       1.00000  0.000000E+00       1.00000  0.000000E+00      0.909979       58.3024       1.00000       11.8737  0.000000E+00       2.22009  0.000000E+00       2.22009  0.000000E+00  0.000000E+00      -4.44018       58.0434     -0.634584        1       Scatag_D
      92       613.064       3493.70  0.000000E+00  0.000000E+00       2058.71  0.000000E+00       1411.57       1492.27  0.000000E+00       5786.56       29.6501       8127.13 -1.000000E+10 -1.000000E+10 -1.000000E+10 -1.000000E+10 -1.000000E+10  0.000000E+00  0.000000E+00  0.000000E+00  0.000000E+00  0.000000E+00  0.000000E+00  0.000000E+00  0.000000E+00  0.000000E+00  0.000000E+00  0.000000E+00  0.000000E+00       Funtag_D
      93  1.574339E+05  1.344910E+05       22943.0       12.7645      0.281145       1.90741      0.256502      0.255217    -0.0829918    -0.0865613    0.00467192      0.799919       Opttag_D
      93       61.2824       6.02516       1.00000  0.000000E+00       1.00000  0.000000E+00      0.910095       58.3064       1.00000       11.8802  0.000000E+00       2.21792  0.000000E+00       2.21792  0.000000E+00  0.000000E+00      -4.43584       58.0434     -0.634584        1       Scatag_D
      93       610.197       3482.38  0.000000E+00  0.000000E+00       2055.74  0.000000E+00       1408.51       1482.56  0.000000E+00       5773.99       29.1213       8100.48 -1.000000E+10 -1.000000E+10 -1.000000E+10 -1.000000E+10 -1.000000E+10  0.000000E+00  0.000000E+00  0.000000E+00  0.000000E+00  0.000000E+00  0.000000E+00  0.000000E+00  0.000000E+00  0.000000E+00  0.000000E+00  0.000000E+00  0.000000E+00       Funtag_D
      94  1.573851E+05  1.344939E+05       22891.2       10.6901      0.281501       1.91704      0.256512      0.255251    -0.0830169    -0.0866546    0.00467038      0.798933       Opttag_D
      94       61.3049       6.03173       1.00000  0.000000E+00       1.00000  0.000000E+00      0.910181       58.3090       1.00000       11.8853  0.000000E+00       2.21617  0.000000E+00       2.21617  0.000000E+00  0.000000E+00      -4.43234       58.0434     -0.634584        1       Scatag_D
      94       609.793       3473.80  0.000000E+00  0.000000E+00       2053.52  0.000000E+00       1405.28       1470.81  0.000000E+00       5764.63       28.9239       8084.47 -1.000000E+10 -1.000000E+10 -1.000000E+10 -1.000000E+10 -1.000000E+10  0.000000E+00  0.000000E+00  0.000000E+00  0.000000E+00  0.000000E+00  0.000000E+00  0.000000E+00  0.000000E+00  0.000000E+00  0.000000E+00  0.000000E+00  0.000000E+00       Funtag_D
      95  1.573388E+05  1.344955E+05       22843.2       10.2628      0.281885       1.92850      0.256520      0.255281    -0.0830310    -0.0867447    0.00466914      0.798075       Opttag_D
      95       61.3305       6.03868       1.00000  0.000000E+00       1.00000  0.000000E+00      0.910279       58.3120       1.00000       11.8914  0.000000E+00       2.21422  0.000000E+00       2.21422  0.000000E+00  0.000000E+00      -4.42845       58.0434     -0.634584        1       Scatag_D
      95       609.469       3466.34  0.000000E+00  0.000000E+00       2050.96  0.000000E+00       1401.06       1463.73  0.000000E+00       5753.90       28.9442       8068.84 -1.000000E+10 -1.000000E+10 -1.000000E+10 -1.000000E+10 -1.000000E+10  0.000000E+00  0.000000E+00  0.000000E+00  0.000000E+00  0.000000E+00  0.000000E+00  0.000000E+00  0.000000E+00  0.000000E+00  0.000000E+00  0.000000E+00  0.000000E+00       Funtag_D
      96  1.573017E+05  1.345008E+05       22800.8       11.8263      0.282371       1.94561      0.256541      0.255325    -0.0830768    -0.0868622    0.00466802      0.797323       Opttag_D
      96       61.3598       6.04743       1.00000  0.000000E+00       1.00000  0.000000E+00      0.910397       58.3152       1.00000       11.8989  0.000000E+00       2.21263  0.000000E+00       2.21263  0.000000E+00  0.000000E+00      -4.42527       58.0434     -0.634584        1       Scatag_D
      96       609.178       3459.81  0.000000E+00  0.000000E+00       2048.36  0.000000E+00       1397.62       1457.61  0.000000E+00       5742.06       29.1008       8057.12 -1.000000E+10 -1.000000E+10 -1.000000E+10 -1.000000E+10 -1.000000E+10  0.000000E+00  0.000000E+00  0.000000E+00  0.000000E+00  0.000000E+00  0.000000E+00  0.000000E+00  0.000000E+00  0.000000E+00  0.000000E+00  0.000000E+00  0.000000E+00       Funtag_D
      97  1.572649E+05  1.345058E+05       22759.2       8.85402      0.282774       1.95945      0.256561      0.255357    -0.0831192    -0.0869492    0.00466583      0.796504       Opttag_D
      97       61.3849       6.05509       1.00000  0.000000E+00       1.00000  0.000000E+00      0.910501       58.3187       1.00000       11.9048  0.000000E+00       2.21136  0.000000E+00       2.21136  0.000000E+00  0.000000E+00      -4.42272       58.0434     -0.634584        1       Scatag_D
      97       608.607       3452.71  0.000000E+00  0.000000E+00       2046.28  0.000000E+00       1395.77       1445.60  0.000000E+00       5732.86       29.1853       8048.18 -1.000000E+10 -1.000000E+10 -1.000000E+10 -1.000000E+10 -1.000000E+10  0.000000E+00  0.000000E+00  0.000000E+00  0.000000E+00  0.000000E+00  0.000000E+00  0.000000E+00  0.000000E+00  0.000000E+00  0.000000E+00  0.000000E+00  0.000000E+00       Funtag_D
      98  1.572263E+05  1.345131E+05       22713.2       8.92719      0.283371       1.97749      0.256586      0.255401    -0.0831828    -0.0870664    0.00466238      0.795275       Opttag_D
      98       61.4230       6.06671       1.00000  0.000000E+00       1.00000  0.000000E+00      0.910659       58.3250       1.00000       11.9138  0.000000E+00       2.20926  0.000000E+00       2.20926  0.000000E+00  0.000000E+00      -4.41852       58.0434     -0.634584        1       Scatag_D
      98       607.707       3442.05  0.000000E+00  0.000000E+00       2043.21  0.000000E+00       1394.82       1437.47  0.000000E+00       5720.51       29.0978       8038.33 -1.000000E+10 -1.000000E+10 -1.000000E+10 -1.000000E+10 -1.000000E+10  0.000000E+00  0.000000E+00  0.000000E+00  0.000000E+00  0.000000E+00  0.000000E+00  0.000000E+00  0.000000E+00  0.000000E+00  0.000000E+00  0.000000E+00  0.000000E+00       Funtag_D
      99  1.571933E+05  1.345193E+05       22674.0       14.0551      0.284102       1.99451      0.256612      0.255450    -0.0832360    -0.0872000    0.00466098      0.793749       Opttag_D
      99       61.4716       6.08071       1.00000  0.000000E+00       1.00000  0.000000E+00      0.910855       58.3307       1.00000       11.9264  0.000000E+00       2.20573  0.000000E+00       2.20573  0.000000E+00  0.000000E+00      -4.41146       58.0434     -0.634584        1       Scatag_D
      99       607.341       3428.86  0.000000E+00  0.000000E+00       2039.44  0.000000E+00       1394.65       1438.48  0.000000E+00       5706.64       28.8276       8029.75 -1.000000E+10 -1.000000E+10 -1.000000E+10 -1.000000E+10 -1.000000E+10  0.000000E+00  0.000000E+00  0.000000E+00  0.000000E+00  0.000000E+00  0.000000E+00  0.000000E+00  0.000000E+00  0.000000E+00  0.000000E+00  0.000000E+00  0.000000E+00       Funtag_D

   MaxLik Scaling Cycle 0105

   Wilson plot

   Statistics in resolution bins after refinement cycle 0003
```


|  |  |
| --- | --- |
|  | - Rxpct factors plot- Rxpct factors table- <Log-Lik Gain>- Ln Sigma A- Correlation Coefficients |


```
   Iters     Tot_Funct    Xray_Funct    Geom_Funct          Grms     rmsD2init     maxD2init             R         Rfree           LLG       LLGfree     rms(bond)    rms(angle)
   Iters             K   B_IMPF_FRAG        K_MISS   B_TEMP_MISS   K_IMPF_MISS   B_IMPF_MISS        K_SOLV   B_TEMP_SOLV   K_IMPF_SOLV   B_IMPF_SOLV        B_TEMP     B_TEMP_11     B_TEMP_12     B_TEMP_22     B_TEMP_13     B_TEMP_23     B_TEMP_33      K_WILSON      B_WILSON      FGCYCLES
   Iters          BOND         ANGLE      IMPROPER       TORSION    SINTORSION      TRIGONAL        PLANAR  BCORRELATION       CONTACT     IDEALDIST        CHIRAL    SIMILARITY       OCCSSUM  UTILDISTANCE     UTILANGLE   UTILTORSION        RHOFIT        ADP-01        ADP-02        ADP-03        ADP-04        ADP-05        ADP-06        ADP-07        ADP-08        ADP-09        ADP-10      RHOFITPC            QM
     100  1.571572E+05  1.345214E+05       22635.8       9.06870      0.284546       2.00186      0.256620      0.255473    -0.0832541    -0.0872646    0.00465765      0.792758       Opttag_D
     100       61.5032       6.08935       1.00000  0.000000E+00       1.00000  0.000000E+00      0.910979       58.3341       1.00000       11.9348  0.000000E+00       2.20315  0.000000E+00       2.20315  0.000000E+00  0.000000E+00      -4.40630       58.0434     -0.634584        1       Scatag_D
     100       606.474       3420.31  0.000000E+00  0.000000E+00       2037.09  0.000000E+00       1394.74       1426.48  0.000000E+00       5698.83       28.6142       8023.24 -1.000000E+10 -1.000000E+10 -1.000000E+10 -1.000000E+10 -1.000000E+10  0.000000E+00  0.000000E+00  0.000000E+00  0.000000E+00  0.000000E+00  0.000000E+00  0.000000E+00  0.000000E+00  0.000000E+00  0.000000E+00  0.000000E+00  0.000000E+00       Funtag_D
   Geometry Restraint Deviations SCREEN Information

 

 The refinement has NOT converged.

 No -bcorrel_zeroocchydrogens_factor flag found - keeping default: will set B for OCC=0 hydrogens to    1.200 times heavy-atom B


 TLS information. Total number of groups:     2


 Group   Natoms    FixRB   FixTLS   Group Tag    Group Spec
     1     2768      Y        Y     tls1    { A|* }
     2     2758      Y        Y     tls2    { B|* }
 
NOTE BUSTER_TLS_SET tls1 { A|* }
NOTE BUSTER_TLS_T   tls1     -1.24008E-01 -8.75701E-03  1.95905E-01 -2.54756E-03 -1.97912E-02  4.23354E-03
NOTE BUSTER_TLS_L   tls1      6.25524E-01  9.73647E-01  4.40986E-01 -4.86570E-02  4.82699E-01 -7.20310E-01
NOTE BUSTER_TLS_S   tls1      1.09103E-01 -2.36495E-01  1.88194E-01  1.15882E-01 -1.08713E-01 -1.46902E-01  1.66685E-01  4.93260E-02
NOTE BUSTER_TLS_O   tls1      2.31588E+01 -6.71772E+01  1.06797E+01
NOTE BUSTER_TLS_FIX tls1 ALL
 
NOTE BUSTER_TLS_SET tls2 { B|* }
NOTE BUSTER_TLS_T   tls2     -1.58512E-01 -6.11579E-02  2.48458E-01  1.04689E-02 -3.47117E-02  9.11453E-03
NOTE BUSTER_TLS_L   tls2      4.70351E-01  3.82030E-01  1.72027E-01  2.16840E-01  2.63951E-01  2.62161E-01
NOTE BUSTER_TLS_S   tls2      2.83079E-02  6.71331E-03 -8.95201E-02 -3.17234E-02 -6.69705E-02  1.54405E-02  6.85666E-02 -3.05844E-02
NOTE BUSTER_TLS_O   tls2     -2.22253E+01 -7.89989E+01  3.21640E+00
NOTE BUSTER_TLS_FIX tls2 ALL

 For final position, Geometry function summary
     term                           number     weight       rms        GooF      function     fn/numb
     ================================================================================================
     BOND bond lengths (angs)       5642       2.000      0.0047       0.232       606.47       0.107
     ANGL bond angles (degs)        7656       2.000        0.79       0.473      3420.31       0.447
     TORS split-harmonic tors       1266       0.000       16.26       1.023         0.00       0.000
     SINTOR sinusoidal tors         1266       2.000       16.26                  2037.09       1.609
     PLAN general plane (angs)       958       5.000      0.0108       0.540      1394.74       1.456
     BCORrelations (angs^2)         5642      10.000       0.446       0.159      1426.48       0.253
     CONT (bad contacts)               0       5.000                                 0.00
     IDEAL(ideal-dist contact)      3796       4.000                              5698.83
     CHIRAL (gelly semiharmon)       742       5.000                                28.61
     SIM similarity restraint for NCS and/or TARGET                               8023.24
     OCCSUM restraint on sum of occupanies                                           0.00       as none defined
     DISTAN utility restraints interatomic distance                                  0.00       as none defined
     UTILANGLE utility angle restraints                                              0.00       as none defined
     UTILTOR utility restraints on torsion angles                                    0.00       as none defined
     Peptide omega torsion angles                           2.24 degs - monitor only

 Weighted rms      0.0047         0.793  (temporary rms bond and angle for BUSTER to pick up!)
 Weighted intra-residual rms(bond) for StdProtein (excluding H)      0.0044
 Outputing geometry progress graph in plotmtv format to file:Geom_Res.mtv

 ############### Running final geometry/screen analysis (screen_final.txt)

 s/r gelly normal termination
```

Statistics and scale factors vs. cycle number
```

|  |  |  |
| --- | --- | --- |
|  | - Rxpct factors- <Log-Lik Gain>- GEOM Residuals- Ln Sigma_A_ | - Scale Factors- B Factors- Imperfection Factors |

```
   Final Geometry Information


---


### 2. Final results

Statistics and scale factors vs. cycle number
```

|  |  |  |
| --- | --- | --- |
|  | - Rxpct factors- <Log-Lik Gain>- GEOM Residuals- Ln Sigma_A_ | - Scale Factors- B Factors- Imperfection Factors |

```
   Average Figure of Merit
   Structure Factor Amplitudes


 # normal termination

  Program stopped.
```

closesok message: time spent in tree = 1278.85 (user) 33.83 (sys)

---

# Normal termination

```
 Resources usage summary:
     Time spent in client ...................  1278.86 (user)       33.85 (system)
     Time spent in scripts (    27 calls) ...     2.26 (user)        0.13 (system)
     Time spent in server ...................     0.00 (user)        0.01 (system)

     Max resident memory used (kb) .......... 432968
     Number of page faults .................. 0 (major)    375915 (minor)
     Number of context switches ............. 2289 (niced)    5241 (other)
```
